# Biomimetic Light‐Driven Aerogel Passive Pump for Volatile Organic Pollutant Removal

**DOI:** 10.1002/advs.202105819

**Published:** 2022-02-23

**Authors:** Sarka Drdova, Shanyu Zhao, Marianna Giannakou, Deeptanshu Sivaraman, Natalia Guerrero‐Alburquerque, Anne Bonnin, Robin Pauer, Zhengyuan Pan, Emanuel Billeter, Gilberto Siqueira, Zhihui Zeng, Matthias M. Koebel, Wim J. Malfait, Jing Wang

**Affiliations:** ^1^ Institute of Environmental Engineering ETH Zurich Stefano‐Franscini‐Platz 3 Zürich 8093 Switzerland; ^2^ Laboratory for Advanced Analytical Technologies Swiss Federal Laboratories for Materials Science and Technology Empa, Überlandstrasse 129 Dübendorf 8600 Switzerland; ^3^ Laboratory for Building Energy Materials and Components Swiss Federal Laboratories for Materials Science and Technology Empa, Überlandstrasse 129 Dübendorf 8600 Switzerland; ^4^ Department of Chemistry University of Fribourg Chemin du Musée 9 Fribourg CH‐1700 Switzerland; ^5^ Swiss Light Source Paul Scherrer Institute Villigen CH‐5232 Switzerland; ^6^ Electron Microscopy Center Swiss Federal Laboratories for Materials Science and Technology Empa; Überlandstrasse 129 Dübendorf CH‐8600 Switzerland; ^7^ State Key Laboratory of Pulp and Paper Engineering South China University of Technology Guangzhou 510640 China; ^8^ Department of Chemistry University of Zurich Winterthurerstrasse 190 Zürich CH‐8057 Switzerland; ^9^ Cellulose and Wood Materials Laboratory Swiss Federal Laboratories for Materials Science and Technology Empa, Überlandstrasse 129 Dübendorf 8600 Switzerland; ^10^ School of Materials Science and Engineering Shandong University Jinan 250061 China

**Keywords:** aerogel, manganese oxide, passive pump, thermal transpiration, VOCs degradation

## Abstract

Inspired by the solar‐light‐driven oxygen transportation in aquatic plants, a biomimetic sustainable light‐driven aerogel pump with a surface layer containing black manganese oxide (MnO_2_) as an optical absorber is developed. The flow intensity of the pumped air is controlled by the pore structure of nanofilbrillated cellulose, urea‐modified chitosan, or polymethylsilsesquioxane (PMSQ) aerogels. The MnO_2_‐induced photothermal conversion drives both the passive gas flow and the catalytic degradation of volatile organic pollutants. All investigated aerogels demonstrate superior pumping compared to benchmarked Knudsen pump systems, but the inorganic PMSQ aerogels provide the highest flexibility in terms of the input power and photothermal degradation activity. Aerogel light‐driven multifunctional gas pumps offer a broad future application potential for gas‐sensing devices, air‐quality mapping, and air quality control systems.

## Graphical abstract

Porous aerogels facilitate a passive gas flow under an applied thermal gradient based on the thermal transpiration process. This study demonstrates the operation of a bilayer aerogel pump with a top layer containing a light absorber with catalytic properties. Therefore, irradiation is used as a sole power input to pump and degrade airborne organic pollutants simultaneously.

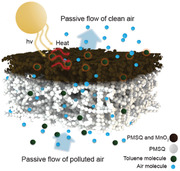

## 1 Introduction

Exposure to volatile organic pollutants (VOCs) poses one of the leading environmental and health risks and raise the need to develop sustainable solutions to monitor and remove airborne organic pollutants. The existing technology readiness for gaseous pollutants treatment is based mainly on adsorption, ozonation, and photocatalytic degradation.^[^ ^1^ ^]^ Among them, photocatalytic degradation is of great interest, particularly for low‐concentration pollutants, as the photocatalyst ultimately decomposes the VOCs to benign final products (CO_2_ and H_2_O).^[^ ^2^ ^]^ However, the widespread adoption of photocatalytic degradation is still restricted by several obstacles. Among others, the process either involves auxiliary power inputs to support the mass transfer (energy intensive) or relies on diffusion‐related absorbent materials (low efficiency).^[^ ^3^ ^]^ In contrast, an ideal solution would rely on a sustainable‐energy (e.g., solar) supported gas transport system that drives the air and pollutant to flow through the catalyst zone, where the pollutant is degraded upon the activation of the catalyst (e.g., under solar irradiation). Such passive gas transport, i.e., thermal transpiration, exists in aquatic plants, where it occurs under solar irradiation owing to the unique pore structure of young leaves.^[^ ^4^ ^]^ Upon irradiation, the temperature gradient induced on the pores initiates the flux of air from the atmosphere to supply oxygen to the root system.

The thermal transpiration process was first explored by Knudsen in 1909 using long glass tube capillaries.^[^ ^5^ ^]^ The induced temperature gradient resulted in the one‐way gas flow by heating one end of these capillaries. However, the operation of thermal transpiration at ambient pressure and temperature has seen only limited development until the 21st century. This is due to the requirement for materials with channels that are comparable in size to the mean free path of gas molecules (≈70 nm at 25 °C, 1 atm). Modern adaptations of thermal transpiration have been realized by applying selective etching, micromachining or porous membranes containing pristine open channels.^[^ ^6^ ^]^ The porous materials provide a large number of pores with various sizes, which is beneficial for achieving a strong mass flux.^[^ ^7^ ^]^

The general requirements for generating effective thermal transpiration in a porous material are: i) a maintained temperature gradient that is not significantly compromised by a strong gas transport across the porous material, ii) an efficient gas conduction through the open pores, and iii) an appropriate ratio of the mean free path of gas molecules to the pore chord length (characterized by a Knudsen number *K*_n_ in the range of 1–10).^[^ ^8^, ^9^ ^]^ As compared with traditional porous materials, aerogels are ideal candidates for the fabrication of thermal transpiration pumps, also known as Knudsen pumps (KP), owing to their low density, high porosity, and ultralow thermal conductivity.^[^ ^10^, ^11^, ^12^ ^]^ For aerogel‐based KP, the thermal gradient is typically induced by heating one side with electrical resistive heaters that require an additional power input.^[^ ^12^ ^]^ Furthermore, the air gaps between the heater and the porous membrane can impede the pump performance.^[^ ^9^ ^]^ In nature, thermal transpiration in aquatic plants is induced passively by solar energy, one of the most environmentally sustainable power inputs. By imitating this natural system, Young et al.^[^ ^13^ ^]^ developed a 15‐stage aerogel‐based KP device, which produced a mass flow of 3.5 × 10^−9 ^kg s^−1^ of nitrogen with the irradiation of 175 mW cm^2^. However, irradiation on the optically transparent aerogel surface generates very limited thermal energy. Therefore, a dual‐functional (pumping and VOC degradation) aerogel KP prototype was 3D printed with a top layer containing light‐absorbing heterogeneous manganese oxide nanoflakes serving as nanoheater and catalyst with excellent absorption in the full solar spectrum.^[^ ^14^, ^15^, ^16^ ^]^ However, the limited pore size range and complicated pore structure (small pores in the filler phase and large pores in the binder phase)^[^ ^15^ ^]^ limited the capability and use potential of the aerogel KP prototype. Hence, in this study, we fabricate new aerogel KP systems and explore pore formation control to regulate the gas pumping and VOC degradation properties. We demonstrate KP devices composed of various aerogel materials with complementary properties in terms of the pore morphology, accessible pore size, and operation temperature. Three aerogels have been selected based on their pore sizes and morphologies: large pore (average pore size, *D*_pore_ > 0.5 µm) nanofibrillated cellulose (NFC) aerogel,^[^ ^17^ ^]^ medium pore (*D*_pore_ > 150 nm) urea‐modified chitosan (UMCh) aerogel,^[^ ^18^ ^]^ and small pore (*D*_pore_ < 50 nm) organo‐modified siloxane polymethylsilsesquioxane (PMSQ)^[^ ^19^ ^]^ aerogel. Thereby, during the transport of the air through a bilayer aerogel membrane, the removal of organic pollutants is facilitated by the adsorption onto the aerogel porous structure and MnO_2_ surface active sites followed by the photo‐thermocatalytic degradation. Besides the presented application of organic pollutant removal using light as the sole sustainable energy input, many other possible applications can be implemented with the light‐driven KP system. This includes gas transportation, gas sampling and sensing, and toxic compounds, bacteria and viruses removal from polluted air. Therefore, the core innovation presented in our study is the motionless solar‐driven passive pump integrated with multiple potential functions for various possible applications.

## 2 Results and Discussion

**Figure 1:**
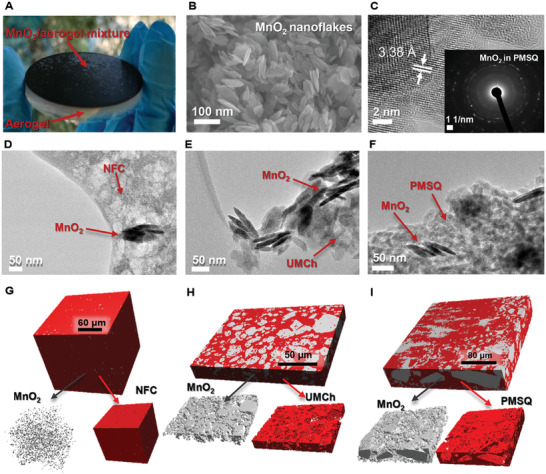
Aerogel membranes implemented with surface‐active absorber layer. Photos of A) polymethylsilsesquioxane (PMSQ) based bilayer aerogels. B) SEM and C) HRTEM image of MnO_2_ nanoflakes with the selected area electron diffraction (SAED) pattern of MnO_2_ embedded in PMSQ. TEM of MnO_2_ nanoflakes embedded in D) nanofibrillated cellulose (NFC), E) urea‐modified chitosan (UMCh), and F) PMSQ aerogels. 3D volume rendering based on X‐ray tomography of the top absorber layer cutout of the low‐density aerogel matrix (red) with shown/separated MnO_2_ nanoflakes (grey, 10 wt%) embedded in G) NFC (178.75 × 178.75 × 157.63 µm^3^, stacks of 1100 cross sections), H) UMCh (211.25 × 1625.00 × 28.11 µm^3^, stacks of 1000 cross sections), and I) PMSQ aerogel matrix (363.68 × 351.00 × 61.10 µm^3^, stacks of 2160 cross sections).

The bilayer aerogel membranes (**Figure** **1**A) were fabricated by mixing 10 or 50 wt% (relative to aerogel dry mass) MnO_2_ nanoflakes (hydrothermally synthesized, ≈100 nm × 10 nm, Figure 1B,C)^[^ ^14^ ^]^ with aerogel sols before triggering gelation (Figure S1, Supporting Information). The PMSQ sol has a low enough viscosity (Figure S2, Supporting Information) to allow the settling of MnO_2_ particles into a compact MnO_2_ rich layer (80 and 300 µm thick for the 10 and 50 wt% samples, respectively, Figures S2–S4, Supporting Information). Higher viscosities of the NFC and UMCh sols (Figure S2, Supporting Information) necessitate a different approach: a bilayer gel has been prepared by casting a MnO_2_‐bearing NFC or UMCh sol onto a previously gelled NFC or UMCh substrate to form a MnO_2_ surface rich layer. The MnO_2_‐rich layers are 2 and 6 mm for NFC (very high viscosity hinders a thin layer formation, Figure 1G), and 200 and 300 µm for UMCh at 10 and 50 wt% MnO_2_ loadings, respectively (Figure S3, Supporting Information). The overall thickness of NFC and UMCh has been controlled in the range of 7–10 mm, whereas the different preparation route of the PMSQ sample provides thinner overall thickness of 5 mm. The prepared membranes are further characterized for MnO_2_ distribution in the absorber layer and evaluated for pumping and VOC (i.e., toluene) decomposition performances.

The distribution of MnO_2_ nanoflakes in the surface absorber layer (Figure 1D–F and Figure S4, Supporting Information) has been retrieved by X‐ray tomography (Figure 1G–I and Figure S5, Supporting Information), revealing the MnO_2_ aggregate sizes in the range of 1–5, 1–14, and 1–50 µm (with the mean values of 2.7, 4.4, and 21 µm) in the NFC, UMCh, and PMSQ aerogels, respectively (Figures S6–S8, Supporting Information). Although large MnO_2_ aggregates are present in both UMCh and PMSQ aerogels (Figure 1H,I), the aggregates with smaller geometric diameters (<10 µm) are dominant in UMCh aerogel as displayed by particle size analysis (Figures S6 and S7, Supporting Information), and scanning electron microscopy (SEM) cross‐section analysis (Figure S5F,G, Supporting Information). The variations of the aggregate size distributions are attributed to the distinct preparation procedures and the nature of the sols^[^ ^19^ ^]^ (Table S1, Supporting Information).

**Figure 2:**
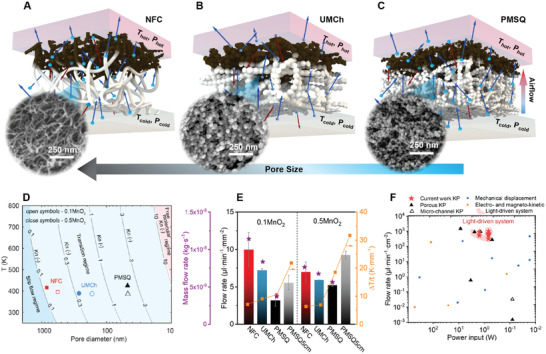
Effect of pore size and structure on pumping performances. Thermal transpiration across A) cellulose nanofibrillar (NFC), B) urea‐modified chitosan (UMCh), and C) polymethylsilsesquioxane (PMSQ) nanoparticulate aerogel structures. White represents the neat aerogel structure; brown represents the aerogel matrix with embedded MnO_2_. The blue and red arrows depict the air molecules’ movement. D) Contour plots of *K*_n_ as a function of pore diameter and temperature at 1 bar of air pressure. The test conditions for the NFC, UMCh, and PMSQ membranes are indicated by symbols. E) Pumping performance indicated by the mass and volumetric flow rates and temperature difference per unit sample thickness (∆*T*/*t*) after irradiation at 20 cm (97 mW cm^2^) and 5 cm (222 mW cm^2^, PMSQ only). F) Flow rate versus power input and comparison with state of the art (Tables S2 and S3, Supporting Information). The performance of reported Knudsen pumps (KP) made from porous materials and lithographic microchannels are highlighted using full and open black triangles, respectively.

To evaluate pumping performances, the functional bilayer aerogel membranes are sealed into a two‐compartment reactor (Figure S9, Supporting Information) and irradiated by a 300 W halogen lamp.^[^ ^15^ ^]^ The high photothermal conversion efficiency of MnO_2_ establishes a temperature difference (∆*T*) between the hot and cold regions, which are separated by the insulating aerogel frameworks with different sub‐µm pore sizes (**Figure** **2**A–C and Figure S10, Supporting Information). According to their calculated pore characteristics [Equations (1) and (2)], NFC aerogel with nanofibrils shows the highest porosity (>98%), relatively large average pore size (568 nm), and a high aspect ratio of the pore structure (Figure 2A). UMCh and PMSQ aerogels consist of nanoparticle agglomerate networks with lower porosity (92% and 91%) and intermediate to small pore sizes (168 and 49 nm) (Figure 2B,C). The *K*_n_ numbers are determined from averaged pore sizes [Equation (3)], and they are 0.13, 0.42, and 1.51 for the NFC, UMCh, and PMSQ aerogels, respectively.

All three aerogel membranes fall into the transitional flow regime (Figure 2D), which is favourable for generating thermal transpiration and high mass flux. In the *K*_n_ number range close to that for free molecular flow, the pore sizes are smaller than the mean free path of molecules, which results in the substantial blockage of gas molecules movement and consequent reduction in flow rate. These conditions are favorable for vacuum pumps and gas separation devices.^[^ ^20^, ^21^ ^]^ On the other hand, the pores with *K*_n_ numbers close to the slip flow regime possess comparable or slightly larger sizes than the mean free path of molecules. This results in the reduction of the blockage, which is favorable for gas delivery applications. However, if the pores are too large, the Knudsen effect is reduced and the flow rate also decreases due to the improper ratio between the gas mean free path and the pore size.^[^ ^9^ ^]^ Therefore, an intermediate range of pore sizes, i.e., Knudsen numbers, exists for the maximum flow rate. When we compare the NFC, UMCh, and PMSQ aerogels with 10 wt% MnO_2_, the flowrate of the PMSQ aerogel is the lowest (Figure 2E). The small average pore size of PMSQ aerogel (Figure 2D) and the related restriction of gas molecules transport play the dominant roles in this comparison. For NFC and UMCh, the flow rate decreases with increasing MnO_2_ content from 10 to 50 wt% (Figure 2E), which could be due to the effect of increased average pore sizes and decreased *K*_n_ numbers (Figure 2D and Figure S11, Supporting Information) leading to a less efficient Knudsen effect. In the case of PMSQ aerogel, the average pore size does not change by the increase of MnO_2_ content (Figure 2D) while the temperature gradient increases. This may explain the stronger flow rate. Another caveat is that the actual pores in the membranes have certain distributions, while the average pore size is considered in the above qualitative discussion.

The irradiation of 97 mW cm^−2^ on the membrane surface is achieved for a lamp distance of 20 cm (equivalent to 0.7 sun referring to an annual average of solar irradiation). The irradiation has increased to 222 mW cm^−2^ (1.6 sun) at 5 cm; however, this is only feasible for the PMSQ sample, which demonstrates high enough thermal stability to stay intact under such a strong irradiation. During the irradiation, the low thermal conductivity (*λ* = 35.7 ± 2.0, 27.9 ± 2.5, and 18.5 ± 2.0 mW m^−1^ K^−1^ for NFC, UMCh, and PMSQ at 298.15 K, 50% relative humidity, and 1 atm) results in the thermal difference per unit membrane thickness (∆*T*/*t*) of 7, 9, and 11 K mm^−1^ at 20 cm irradiation distance for NFC, UMCh, and PMSQ, respectively, and 22 K mm^−1^ at 5 cm irradiation distance for PMSQ (Figure 2E). As a result, the steady‐state flow rates of 10.0, 7.2, and 3.2 µL min^−1^ mm^−2^ have been obtained for the NFC, UMCh, and PMSQ aerogels with 10 wt.% of MnO_2_ loading (Figure 2E and Figure S12, Supporting Information). Restriction of the flow through the small pores of the PMSQ membrane (compared to NFC and UMCh) can be offset by increasing the MnO_2_ loading to 50 wt% and the irradiation to 222 mW cm^− 2^ resulting in a 9.3 µL min^−1^ mm^−2^ flow rate (Figure 2E). This performance comparable to large‐pore‐size NFC aerogel is facilitated by the enhancement in the photothermal energy conversion resulting in ∆*T*/*t* increase (up to 32 K mm^−1^ for the loading of 50 wt% and irradiation of 222 mW cm^−2^).

**Figure 3:**
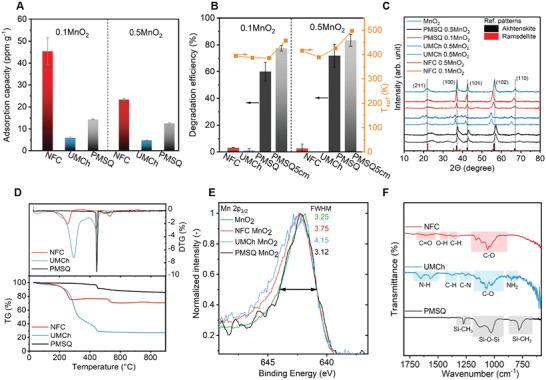
Pollutant degradation performance of the dual‐functional aerogel membranes. A) Adsorption capacity and B) degradation efficiency of toluene and steady‐state temperature on the irradiated surface (*T*_HOT_). C) X‐ray diffraction (XRD) patterns of NFC, UMCh, and PMSQ samples with 10 and 50 wt% of MnO_2_. D) Thermogravimetric analysis (TG and derivative TG) of pristine aerogel materials. XPS spectra of the absorber layer (50 wt% MnO_2_): E) Mn2p_3/2_ electron energy overlay of neat MnO_2_ and MnO_2_ embedded in aerogels, and a list of full width at half maximum. F) FTIR spectra of pure PMSQ, UMCh, and NFC aerogels (more details in Figure S17, Supporting Information).

In brief, although the NFC membrane displays the highest flow rate owing to large pores, the PMSQ membrane allows for stronger irradiances due to its superior thermal stability (**Figure** **3**D). In terms of power consumption, our aerogel gas pumps deliver sustainable pumping flow rates around 3.2–10.0 µL min^−1^ mm^−2^ at a solar power input of 0.69–1.57 W, and they exhibit improved performance compared to reported Knudsen pumps ^[^ ^9^ ^]^ as well as traditional mechanical displacement, electro‐ or magneto‐kinetic micropumps (Figure 2F). The superior pumping performances can lead to an efficient mass transfer of gas pollutants (e.g., VOCs) into the porous aerogels and subsequently toward the catalytically active sites of the absorber layer as demonstrated in the following section.

Two processes occur during VOCs removal using aerogel KP systems: VOCs adsorption onto the porous aerogels and/or VOCs catalytic oxidation. Therefore, the bilayer membranes have been tested for the adsorption and degradation of gaseous toluene as a model pollutant (Figure S9, Supporting Information). The MnO_2_‐PMSQ membrane displays an adsorption capacity of 14.9 ± 0.2 ppm g^−1^ at 298.15 K and a high toluene degradation efficiency (60% at 20 cm and 83% at 5 cm irradiation distance) after 60 min of irradiation exposure (Figure 3A,B). Surprisingly, the MnO_2_‐NFC and MnO_2_‐UMCh aerogels with the same amount MnO_2_ loadings show almost no degradation at the 20 cm irradiation distance (irradiation intensity of 97 mW cm^− 2^, Figure 3A,B). Hence, for PMSQ, the removal of toluene is based on the synergistic effect of adsorption due to the large specific area of the porous aerogel and the photo‐thermocatalytic activity of MnO_2_. The thermocatalytic degradation of VOCs using MnO_2_ nanoflakes is facilitated by photothermal conversion in the whole solar spectrum (Figure S13, Supporting Information), which promotes the activity of lattice oxygen for the catalytic oxidation.^[^ ^14^, ^22^ ^]^ While the removal of VOCs by NFC and UMCh relies on the sole adsorption of the porous structures, the organic aerogel materials might interact with MnO_2_ surface and inhibit its reactivity towards toluene degradation. The organic membranes show the adsorption capacities of 54 ± 6 and 6.4 ± 0.4 ppm g^−1^ for NFC and UMCh with 10 wt% MnO_2_ loading at 298.15 K. NFC adsorption capacity is significantly larger compared to UMCh due to the larger specific surface area and the porous structure with larger and more accessible pores (Figure S14C, Supporting Information). As compared to the pristine aerogels, the NFC and UMCh composites with 50 wt% MnO_2_ loadings show reduced specific surface area, and lower amount of accessible pores, which subsequently reduce the adsorption capacities. However, the applied MnO_2_ layer does demonstrate obvious improvements for pumping and VOC removal performances (Figures S12 and S14, Supporting Information). In conclusion, for MnO_2_‐PMSQ membrane, the combination of the light‐driven VOCs transport, adsorption and degradation has great potential for a sustainable indoor air cleaning. However, MnO_2_‐NFC and MnO_2_‐UMCh membranes possess only the qualities for light‐driven passive gas sorption (collectors) or sole gas transport applications.

To characterize the MnO_2_‐aerogel composites and to shed light on the mechanism of the MnO_2_ reactivity and its inhibition, all three membranes were analyzed and compared using X‐ray diffraction (XRD), X‐ray photoelectron spectroscopy (XPS), hard X‐ray photoelectron spectroscopy (HAXPES), and Fourier‐transformed infrared (FTIR) spectroscopy. The neat MnO_2_ is identified by XRD as a heterogeneous phase consisting of hexagonal akhtenskite (*ε*‐MnO_2_) and orthorhombic ramsdellite structures (*γ*‐MnO_2_).^[^ ^16^, ^23^ ^]^ XRD further reveals no significant changes of the original MnO_2_ material after incorporating into aerogel membranes as no new peaks are observed (Figure 3C and Figure S15, Supporting Information) (the systematic shifts in the diffraction pattern positions are attributed to the specimen displacement during the XRD analysis). In contrast, the surface‐sensitive XPS analysis^[^ ^24^ ^]^ reveals changes in the Mn 2p_3/2_ core‐level spectra (Figure 3E), indicating the modification of the surface environment of MnO_2_, i.e., oxidation state and/or local chemical and physical environment.^[^ ^25^, ^26^ ^]^ The Mn 2p_3/2_ peaks of the NFC and UMCh samples display an increase of the full width at half maximum (FWHM) toward higher binding energies (BE) (Figure 3E). On the contrary, only marginal changes in the average oxidation states (AOS) are observed from the applied empirical fitting model for Mn 2p_3/2_ peak deconvolution (3.4+ for NFC, 3.7+ UMCh, and 3.8+ PMSQ comparing to the neat MnO_2_ AOS of 3.5+ and 3.7+ from HAXPES, Figure S16, Supporting Information).^[^ ^27^ ^]^ The variations in the peak width and location could be, therefore, attributed to the interaction of MnO_2_ with the surrounding functional carboxylate and ureido groups originated from the chemical components of NFC and UMCh as shown by FTIR analysis (Figure 3F and in details Figure S17, Supporting Information).^[^ ^26^ ^]^ Together, the shifts in XPS and FTIR spectra, and the large magnitude of the zeta potential of MnO_2_ in the NFC and UMCh sols (Table S1, Supporting Information) demonstrate the interactions with the functional groups of NFC and UMCh perturb the surface electronic structure of MnO_2_.^[^ ^28^, ^29^ ^]^ As a result, the redox catalytic degradation of VOCs is hindered due to the limited active sites accessibility for gaseous pollutants.^[^ ^30^ ^]^ In contrast, the XPS peak of Mn 2p_3/2_ in the PMSQ sample resembles the Mn 2p_3/2_ of the neat MnO_2_ indicating a limited change of the surface electronic structure of MnO_2_ after the incorporation into the PMSQ matrix, and thus, the least disturbance on the catalytic degradation of toluene (Table S4, Supporting Information). In addition, SEM observation of the bilayer membrane surfaces after the irradiation reveals morphological changes for NFC and UMCh (Figure S18, Supporting Information). Although the membranes are macroscopically stable without a significant observed shrinkage, the microscopic structure of NFC and UMCh display signs of annealing and partial merging of aerogel particles on the surface due to the high local temperature spots. The merging of aerogel particles with MnO_2_ nanoflakes may contribute to the disturbance of MnO_2_ catalytic activity.

**Figure 4:**
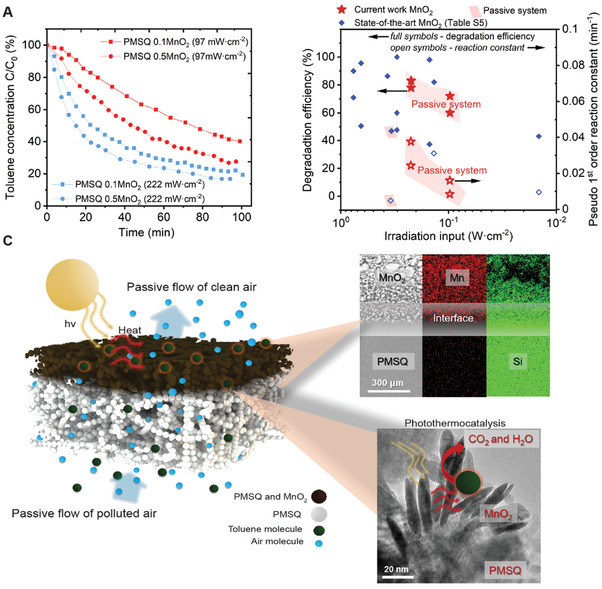
Toluene degradation performance of MnO_2_‐PMSQ membrane. A) Toluene concentration evolution as a function of the irradiation time. B) Comparison of the state‐of‐the‐art for VOC degradation efficiency versus irradiation intensity (Table S5, Supporting Information). C) Solar‐driven thermal transpiration with simultaneous photothermal catalytic VOC degradation (left panel) with scanning electron microscopy (SEM) and energy dispersive X‐ray analysis (EDX) images of MnO_2_‐PMSQ and PMSQ aerogel interface (right upper panel), and representation of photothermal conversion on MnO_2_ nanoflakes (right lower panel).

Motivated by the superior pumping and toluene removal performance of the PMSQ sample, the effect of irradiation and MnO_2_ loading is further investigated in terms of the degradation efficiency and rate constant [Equations (5)–(7)] (**Figure** **4**A–C). The amount of adsorbed photons, temperature and catalytic activity can be enhanced by intensifying the light power and/or by increasing the MnO_2_ loading (Figure 4A). The maximum rate constant of 0.0415 min^−1^ [Figure 4C and Equation (6)] has been achieved for the 50 wt% MnO_2_‐PMSQ sample with 222 mW cm^−2^ irradiation. More intense irradiation induces a stronger thermal reaction by the activation of lattice oxygen,^[^ ^31^, ^32^ ^]^ and it generates more reactive oxygen species (∙O^2−^).^[^ ^33^ ^]^ Moreover, higher MnO_2_ loading provides more active sites that interact with pollutants. Nevertheless, the improvement of the degradation efficiency by adding MnO_2_ has its limit and it saturates at a certain loading, at which the shading effect plays a significant role.^[^ ^34^ ^]^ In addition, the SEM analysis reveals that the highest concentration of MnO_2_ is located 10–70 µm below the membrane surface (Figure 4C, upper panel), which may leave room for further optimization of the photothermal conversion and VOC degradation. Despite this, our results show a similar or better degradation performance per unit irradiation input in comparison to reported photoactivated MnO_2_ based catalysts (Figure 4B). Furthermore, the MnO_2_‐PMSQ membrane demonstrates better toluene degradation in contrast to a 3D printed aerogel membrane,^[^ ^15^ ^]^ which only provides ≈50% degradation (and rate constant of 0.0042 min^−1^) for the same irradiation time. The preliminary cyclical test shows that the membrane maintains the pumping performance, but the toluene degradation is reduced by 45% in the second cycle, mainly due to the catalyst poisoning effect (Figure S19, Supporting Information). Hence, the minimization of catalyst deactivation would be the future focus for the successful implementation of the membrane. Our data provide a proof‐of‐concept of a simultaneous light‐driven pumping and photocatalytic degradation of toluene (or other potential organic compounds based on previous studies ^[^ ^14^, ^23^, ^35^ ^]^) in one single composite material, assisted by light irradiation but without other extra power input or moving parts, meaning no wear or noise. Moreover, the concept is scalable and can be further optimized with respect to the distribution of the catalyst particles and by using other efficient light adsorbents, such as black TiO_2_.

## 3 Conclusion

In summary, a biomimetic light‐driven passive gas pump has been developed using NFC, UMCh, and PMSQ aerogels with a surface layer containing dual‐functional MnO_2_ nanoflakes. The pumping performance has been found to correlate with *K*_n_ as defined by the average size of pores. The lower flow rate for the PMSQ aerogel can be compensated by increasing the irradiation power or light absorber concentration. The passive aerogel pumps demonstrate an outstanding pumping performance compared to the state‐of‐the‐art miniaturized gas pumps, with potentials for sustainable and inexhaustible solar‐driven applications in air‐quality mapping, gas flow regulation, and gas sensors. Additionally, we demonstrate the PMSQ aerogel with embedded MnO_2_ as a suitable candidate for simultaneous, solar‐driven pumping and photothermal oxidation (i.e., degradation) of organic gaseous pollutants, with the great potential to scale up for sustainable indoor air cleaning.

## 4 Experimental Section

### Manganese Oxide (MnO_2_) Nanoflakes

10.04 g Mn(NO_3_)_2_·4H_2_O (Sigma Aldrich) was dissolved in distilled water (50 wt%). Then, 100 mL of a 3.06 wt% KMnO_4_ aqueous solution was added dropwise and stirred at 60 °C for 12 h. The precipitate was filtered and dried at 60 °C overnight.^[^ ^27^ ^]^

### PMSQ‐MnO_2_ Aerogels Membrane Synthesis

20.4 g urea (BioXtra, Sigma Aldrich) was dissolved in 49 mL 5 mmol L^−1^ acetic acid aqueous solution under room temperature (25 °C), after 5 min agitation at 150 rpm, 16.8 mL cetyltrimethylammonium chloride (CTAC, 25 wt% in H_2_O, Sigma Aldrich), and 23.2 mL methyltrimethoxylsilane (97%, ABCR Swiss AG) were added, the agitation was continued at 300 rpm for 30 min, and the solution was stored at 4 °C as a stock sol, but the stock sol was only kept for maximal 2 d before any gel preparation.^[^ ^36^ ^]^ 0.1 g/0.5 g R‐MnO_2_ was added to 10 mL stock sol and speed mixed at 2350 rpm for 1 min, the solution was poured in PS molds (ø 54 × 14 mm, Semadeni, article no. 1709), and placed in a 65 °C oven for 40 h, the gelation occurred after 5 h, which allows the settlement of the MnO_2_ to form a compacted surface layer. The gels were washed four times by distilled water (gel:solvent ratio 1:10, 65 °C, 12 h intervals), and then the solvent water was exchanged three times by ethanol (denatured with 5% isopropyl alcohol, Alcosuisse, Switzerland, gel:solvent ratio 1:10, 65 °C, 12 h intervals). The alcogels were dried in a continuous supercritical extraction process (Separex, France) for 7 h at 50 °C and 120 bar of CO_2_ at 20 g min^−1^.

### NFC‐MnO_2_ Aerogels Membrane Synthesis

2,2,6,6‐Tetramethyl‐1‐piperidinyloxyl (TEMPO)‐mediated oxidation procedure was performed on never‐dried elemental chlorine free bleached cellulose fibers from soft wood pulp (*Picea abies* and Pinus spp.). This pulp was obtained from Stendal GmbH (Berlin, Germany). The procedure used for the oxidation treatment was adapted from the method previously described,^[^ ^37^, ^38^ ^]^ whereby cellulose fibers were initially suspended in water to a final concentration of 2 wt%. TEMPO and NaBr were dissolved in water and mixed with the cellulose pulp suspension at 0.1 and 1.0 mmol g^−1^ cellulose, respectively. The suspension pH was then adjusted to 10 by dropwise addition of 2 wt% NaOH. Finally, NaClO was added at 10 mmol g^−1^ cellulose, and the reaction proceeded under mild agitation at room temperature for 5 h. TEMPO and sodium hypochlorite (NaClO) solution (12%–14% chlorine) were purchased from VWR international. Sodium bromide (NaBr ≥ 99%) and sodium hydroxide (NaOH ≥ 99%) were supplied by Carl Roth GmbH & Co. Next, the TEMPO‐oxidized cellulose fibers were washed several times with distilled water, and subsequently ground using a Supermass Colloider (MKZA10‐20 J CE Masuko Sangyo, Japan) at an applied energy of 9 kWh kg^−1^. The ground cellulose nanofibers were further fibrillated using a high shear homogenizer (M‐110EF, Microfluidics Ind., Newton, MA‐USA) for ten passes at a pressure of 8 bar, yielding a uniform and transparent CNF suspension at a concentration of 0.5 ± 0.1 wt%. The CNF suspension was stored in a refrigerator at 4 °C until further use. This NFC was further mechanically homogenized and sonicated to remove fibril coiling and imperfections and then diluted to 0.7 wt% clear suspension, which was kept in storage at 4 °C. 35/25 mL of this solution was taken in a PS mold (53 × 53 × 30 mm; Semadeni, article no. 2965). In a 185 mL PP container, 5/15 mL of 0.7 wt% NFC was taken and 0.1 g/0.5 g R‐MnO_2_ was added and speed‐mixed for 5 min at 2350 rpm (container and mixer by Hauschild GmbH & Co. KG, Hamm, Germany). This NFC/MnO_2_ precursor was pipetted carefully to PS mold bottom to have a clear bottom layer of NFC/MnO_2_. Gelation was done in an acidic‐vapor phase pH change method, described previously.^[^ ^9^ ^]^ These hydrogels were solvent exchanged in stages to 99% ethanol and dried in a continuous supercritical extraction process (Separex, France) for 6 h at 50 °C and 120 bar of CO_2_ at 20 g min^−1^.

### UMCh‐MnO_2_ Aerogels Membrane Synthesis

8 g chitosan (>400 mPa s for 1% in acetic acid @20 °C) was dissolved in 80 mL of a 1.12 m HCl aqueous solution under constant agitation at 70 °C oil bath for 2 h. The resulting solution was left to cool at room temperature for 5 min under stirring. After dissolution of 18 g of urea, 60 µL CTAC was added, and then the solution was centrifuged at 6000 rpm for 3 min to eliminate the bubbles and solid impurities.^[^ ^39^ ^]^ 0.1 g/0.5 g R‐MnO_2_ was added to 5 mL stock sol and speed mixed at 1250 rpm for 30 s. The chitosan‐MnO_2_ solution was poured in square silicone molds, subsequently, the rest of the chitosan‐urea solution 20 mL were added on top. The mold was enclosed in a bigger plastic polypropylene recipient. Gelation and aging took place at 80 °C for 24 h. The gels were exchanged into ethanol three times (gel:solvent ratio 1:10, 65 °C, 24 h each) and then dried in a continuous supercritical extraction process (Separex, France) for 7 h at 50 °C and 120 bar of CO_2_ at 20 g min^−1^.

### Synchrotron X‐Ray Tomographic Microscopy

3 × 3 × 10 mm rectangular bilayer samples were cut and measured by tomography, which include MnO_2_ surface layer and pristine aerogel layer. Imaging was performed at the TOMCAT beamline of the Swiss Light Source, situated on a 2.9 T bending magnet, and equipped with a multilayer monochromator. X‐ray images were acquired at 12 keV and a propagation distance of 10 mm. The X‐ray indirect detector comprised a LSO:Tb 5.8 µm scintillator, a 40× optical objective, and a sCMOS pco.EDGE camera (6.5 µm pixel size, 2560 × 2160 pixels), resulting in an effective pixel size of 0.162 µm. During the continuous tomography scan, 1501 projections were collected over 180° with an exposure time of 500 ms per projection as well as two series of 50 flats and a series of 20 dark projections. The data were reconstructed using the Gridrec algorithm^[^ ^28^ ^]^ with and without prior phase‐retrieval (Paganin, delta = 6.3e^−6^, beta = 3.6e^−8^ with unsharp mask of 0.3 width and sigma = 1).

### 3D Image Analysis

X‐ray images were imported into ImportGeo‐Vol (GeoDict, Math2Market) for reconstructing the samples. The voxel sizes were 0.1625 µm, corresponding to the effective pixel sizes used during the tomography scan. After a series of image processing steps, the Otsu Single Threshold method was used for segmenting MnO_2_ from the background. Then volume fractions of aerogel matrix were calculated based on the reconstructed samples. The size distributions of R‐MnO_2_ nanoflakes were analyzed with MatDict (GeoDict, Math2Market), and the diameter of a R‐MnO_2_ nanoflake was defined as the largest sphere that fit into the envelope of this nanoflake.

### Rheology

Rheological properties of the NFC, UMCh, and PMSQ sols at different temperatures 20, 30, 40, 60, and 70 °C were recorded on a rotational rheometer (MCE502, Anton Paar) with a coaxial cylinder geometry, according to the ISO 3219:1995 Standard. Apparent viscosities were measured at different temperatures via steady‐state flow with a sweep of shear rate from 0.1 to 100 s^−1^. The sols were equilibrated for 60 s before testing.

### Zeta Potential

Zeta potential was measured on diluted sols of ≈0.1 wt%, MnO_2_ in the solvents (0.1 wt%), and MnO_2_ in relating sols (0.1 wt% of the solvents), respectively, using a Zetasizer NanoZS instrument (Malvern, UK). The zeta potentials were calculated from electrophoretic mobility using a formula valid for spherical particles, and the data were used for comparative purpose only.

### Thermal Conductivity

Thermal conductivities were determined from separately prepared square plate specimens (around 48 × 48 × 7 mm^3^, cast simultaneously from the same sols) of monolithic aerogels using a custom‐built guarded hot plate device (guarded zone: 50 × 50 mm^2^, measuring zone: 25 × 25 mm^2^) designed for small samples of low thermal conductivity materials with a 10 °C temperature difference. In order to be consistent with measurements according to European Standards,^[^ ^29^ ^]^ calibration measurements were carried out using conventional expanded polystyrene (EPS) samples measured once in a 50 cm × 50 cm device, which was then calibrated and validated for the smaller testing equipment. The small guarded hot plate measurement data were then again measured for the same EPS sample in the smaller scale tested device.

### Thermal Stability

Differential thermogravimetric analysis was conducted on a TGA7 analyzer (Perkin Elmer, USA) with the aim to identify the onset temperature to the degradation of organic aerogels and loss of hydrophobic methylsilane groups in the PMSQ aerogel that represent the upper boundary for operation temperature.

### Microstructural Analysis

SEM images were recorded on a FEI Nova NanoSEM 230 (FEI) at an accelerating voltage of 10 kV and a working distance of ≈5 mm. Nominally 15 nm of Pt (measured with a flat piezo detector) was coated to avoid charging, but the effective thickness on the aerogels, with their extreme topography and surface area, will be much lower. Transmission electron microscopic (TEM) images and elemental mappings were recorded by HRTEM (Gatan Ultrascan)/STEM (BF and UDF detector) and EDX (JEOL) using the JEOL JEM2200FS microscope operating at 200 kV. Prior to TEM analysis powder of the sample was prepared and dispersed in methanol, and a drop of finely dispersed sample was put on a Lacey Carbon film copper TEM grid. The TEM grid with the sample droplet was dried onto a heating plate at 80 °C. NFC sample: The produced 5 cm^3^ of the 0.5 g‐MnO_2_‐NFC aerogel was dissolved in 50 mL of 3 m NaOH using a magnetic stirrer at room temperature for 1 h. 4–5 drops of this black/dark grey solution were dropped on a carbon grid and allowed to dry on a heating plate at 80 °C. This sample was then analyzed as the NFC‐MnO_2_ aerogel sample.

Nitrogen sorption analysis was carried out on a TriFlex nitrogen sorption analyzer (Micromeritics) after prior degassing for 20 h at 100 °C and 0.02 mbar. The specific surface areas (S_BET_, uncertainty ≈50 m^2^ g^−1^) were obtained using the BET method.^[^ ^40^ ^]^ The pore volume (*V*_0_) and average pore diameter (*d*_0_) were calculated from the density of the printed aerogels and their S_BET_ using Equations (1) and (2), respectively^[^ ^41^ ^]^

(1) $$V_{0}=\frac{1}{\rho }\;-\frac{1}{\rho_{\mathrm{skeleton}}}$$

(2) $$d_{0}=\frac{4V_{\mathrm{pore}}}{S_{\mathrm{BET}}}\;$$

where *ρ* is bulk density, *ρ*_skeleton_ is skeletal density, and S_BET_ is specific surface area.

### X‐Ray Diffraction Analysis

The X‐ray diffraction analysis of MnO_2_ powder and the top layer of MnO_2_‐PMSQ, MnO_2_‐NFC, and MnO_2_‐UMCh composites were recorded on a PANalytical X'Pert PRO diffractometer (PANalytical, Netherlands) equipped with a Johansson monochromator (Cu K*α*1 radiation, *λ* = 1.5406 Å).

### Thermal Transpiration Evaluation

The *K*_n_ was calculated from the mean free path of the gas (*l*_m_) and pore chord length (*d*_0_)

(3) $$K_{n}=l_{m}\;/d_{0}$$

in which *l*_m_ was calculated from the average temperature (*T*_avg_) measured at the surface of an aerogel with the lamp distance of 20 or 5 cm, standard pressure (*P*), and gas particle diameter (*d*_g_)

(4) $$l_{m}=k_{B}\;T_{\mathrm{avg}}/\sqrt{2}\pi d_{g}^{2}P$$

where *k*_B_ is the Boltzmann constant and *d*_g_ is 3.711 × 10^−10^ m for gas nitrogen.^[^ ^42^ ^]^ It should be noted that the pressure difference was below the detection limit of the available measurement device (PX409 Series Pressure Transducer, Model No. PX409‐10WDWUUSBH, Omega).

### Ultraviolet–Visible–Near‐Infrared (UV–Vis–NIR) Spectrophotometry

Transmittance and reflectance of the R‐MnO_2_‐aerogel absorber layers and R‐MnO_2_ coated on a glass fiber filter as a reference were measured using UV–vis–NIR spectrophotometer (UV‐3600, Shimadzu) with a high‐performance double monochromator over the wavelength range of 300–1500 nm, which was enabled by three detectors [photomultiplier tube (PMT), In‐GaAs, cooled PbS]. BaSO_4_ was used for baseline and, in the case of reflectance, a spectralon was used as reference. Spectral absorbance was calculated as: Absorbance = 1 − (Reflectance + Transmittance).

### FTIR Spectroscopy

An attenuated total reflection‐Fourier transform infrared spectrometer (Agilent 640 FTIR spectrometer, Agilent technologies, Santa Clara, CA) was used for the characterization of aerogel surface in the spectral width ranging from 4000 to 500 cm^−1^.

### XPS and HAXPES

XPS analysis was performed using a PHI Quantes spectrometer (ULVAC‐PHI), as equipped with a conventional low‐energy Al‐K*α* source (1486.6 eV) and a high energy Cr‐K*α* (5414.7 eV) X‐ray source. Both sources are high flux focused monochromatic X‐ray beams that can be scanned across the sample surface to analyze a selected area on the sample surface. The energy scale of the hemispherical analyzer was calibrated according to ISO 15472 by referencing the Au 4f_7/2_ and Cu 2p_3/2_ main peaks (as measured in situ for corresponding sputter‐cleaned, high‐purity metal references) to the recommended BE positions of 83.96 and 932.62 eV, respectively. Charge neutralization during each measurement cycle was accomplished by a dual beam charge neutralization system, using low energy electron and argon ion beams (1 V bias, 20 *μ*A current). XPS survey spectra, covering a BE range from 0 to 1100 eV, were recorded with a step size of 0.25 eV at a constant pass energy of 140 eV using the Al‐K*α* source (power 51 W; beam diameter ≈200 μm). High‐resolution spectra of the main constituents were recorded with a step size 0.13 eV at a constant pass energy of 69 eV. HAXPES survey spectra, covering the same BE range as XPS survey spectra, were recorded with a step size of 0.5 eV at a constant pass energy of 140 eV using the Cr‐K*α* source (power 45.4 W; beam diameter ≈100 μm). High‐resolution spectra of the main constituents were recorded with a step size 0.13 eV at a constant pass energy of 69 eV. Chemical state analysis of each element (i.e., O, C, N, and Si) was performed by constrained peak fitting of the corresponding Shirley background corrected spectra with one or more symmetrical, mixed Gaussian–Lorentzian line shape functions, using the CasaXPS software. The Gauss fraction of each peak component (representative of instrumental broadening) was fixed to 0.85. The Mn peaks were fitted according to an empirical model provided by Ilton et al.^[^ ^27^ ^]^ Atomic concentrations of the elements were calculated from the integrated peak areas using the sensitivity factors provided by the manufacturer (Ulvac‐Phi), as derived according to ISO 18118. Finally, the correction for possible BE shifts due to the incorrect charge neutralization of the sample surface during XPS analysis was required. Thus, the BE scales for each set of measured spectra were aligned by shifting the spectra according to the difference between the BE position of the corresponding C 1s C—H component and the reference value for the C 1s peak of adventitious carbon of 284.8 eV.^[^ ^36^, ^37^ ^]^ For the MnO_2_/NFC composite the C1s C—O component of the cellulose was chosen for BE referencing.^[^ ^38^ ^]^

### Gas Pumping and VOC Degradation

The thermal transpiration and VOC degradation were tested in a stainless steel reactor (Figure S9, Supporting Information). The dried sample was sealed between two compartments of the reactor using a polysiloxane‐based glue (Elastosil RT 601 A and B, Wacker AG). The reactor was equipped with a borosilicate glass window for the light irradiation of the MnO_2_ absorber. The gas flow from the bottom part of the reactor to the outlets in the upper part was driven by the temperature difference generated across the aerogel insulator. The gas flow was monitored using a mass flow meter (MFC, AALBORG TIO Totalizer) and the top and bottom surface temperature of aerogels was measured by a micro‐thermocouple (Type T, Cu‐CuNi). The VOC degradation of the functional micropump was monitored in a closed system containing synthetic air with 25 ppm toluene. First, toluene was introduced into the reactor and circulated using an external pump to reach a steady state condition. After that, the surface of a sample was irradiated using a halogen lamp (300 W) with the light intensity of 97 and 222 mW cm^−2^ (S170C microscope power sensor and Thorlabs PM100USB power/energy meter). The resulting surface temperature increase generated a gas flow through the MnO_2_‐loaded catalytic layer where toluene was degraded. The concentration of toluene was monitored by gas chromatography (GC/FID, Shimadzu GC‐2014). The apparent‐first reaction kinetic model [a simplification of the Langmuir–Hinshelwood (L–H) kinetic model for low‐concentration applications, Equations (5)–(7), where *C_*τ*_* is concentration at time *τ*, *C*_0_ is concentration at *τ* = 0 and *k* is apparent first‐order constant) was applied for the evaluation of the degradation performance^[^ ^16^ ^]^

(5) $$C_{\tau }=C_{0}\;e^{k\tau }$$

(6) $$\ln C_{0}-\ln C_{\tau }=\;k\tau$$

(7) $$E\;\;\left(\%\right)=\frac{C_{0}-C_{\tau }}{C_{0}}\;\cdot 100\%$$

## Conflict of Interest

The authors declare no conflict of interest.

## Author Contributions

S.D. and S.Z. contributed equally to this work. S.D., S.Z., D.S., M.M.K., W.J.M., and J.W. designed the experiments, S.Z., D.S., and N.G. synthesized the PMSQ, NFC, and chitosan aerogels and their MnO_2_ composites, respectively, and characterized SEM and EDX mapping of aerogels. G.S. synthesized the TEMPO‐oxidized nanofibrillated cellulose. S.D. and M.G. prepared the MnO_2_ particles and tested the pumping and VOC degradation, S.D. characterized FTIR and XPS of aerogels. Z.Z. characterized TG and TEM of the NFC aerogels, A.B. collected and Z.P. processed the X‐ray tomography data, R.P. recorded HRTEM and SAED. E.B. conducted the XPS and HAXPES characterization and peak fitting. S.D. wrote the manuscript with the support of S.Z., D.S., W.J.M., and J.W. All authors have given approval to the final version of the manuscript.

## Supporting information

Supporting Information

## Data Availability

The data that support the findings of this study are available from the corresponding author upon reasonable request.
