# Supplementary material for: Biomimetic Light‐Driven Aerogel Passive Pump for Volatile Organic Pollutant Removal
Source: Adv Sci (Weinh). 2022 Feb 23;9(11):2105819. doi: 10.1002/advs.202105819 (PMC9008417; doi:10.1002/advs.202105819)
Supplement: Supplementary file 1 — Supporting Information [file ADVS-9-2105819-s001.pdf]

Supplementary Materials for

Biomimetic light-driven aerogel passive pump for volatile organic pollutant removal

Sarka Drdova†, Shanyu Zhao†,, Marianna Giannakou, Deeptanshu Sivaraman, Natalia Guerrero-Alburquerque, Anne Bonnin, Robin Pauer, Zhengyuan Pan, Emanuel Billeter, Gilberto Siqueira, Zhihui Zeng, Matthias M. Koebel, Wim J. Malfait*, Jing Wang**

† These authors contributed equally to this work.

*Corresponding authors: shanyu.zhao@empa.ch, wim.malfait@empa.ch, jing.wang@ifu.baug.ethz.ch

Supplementary Text

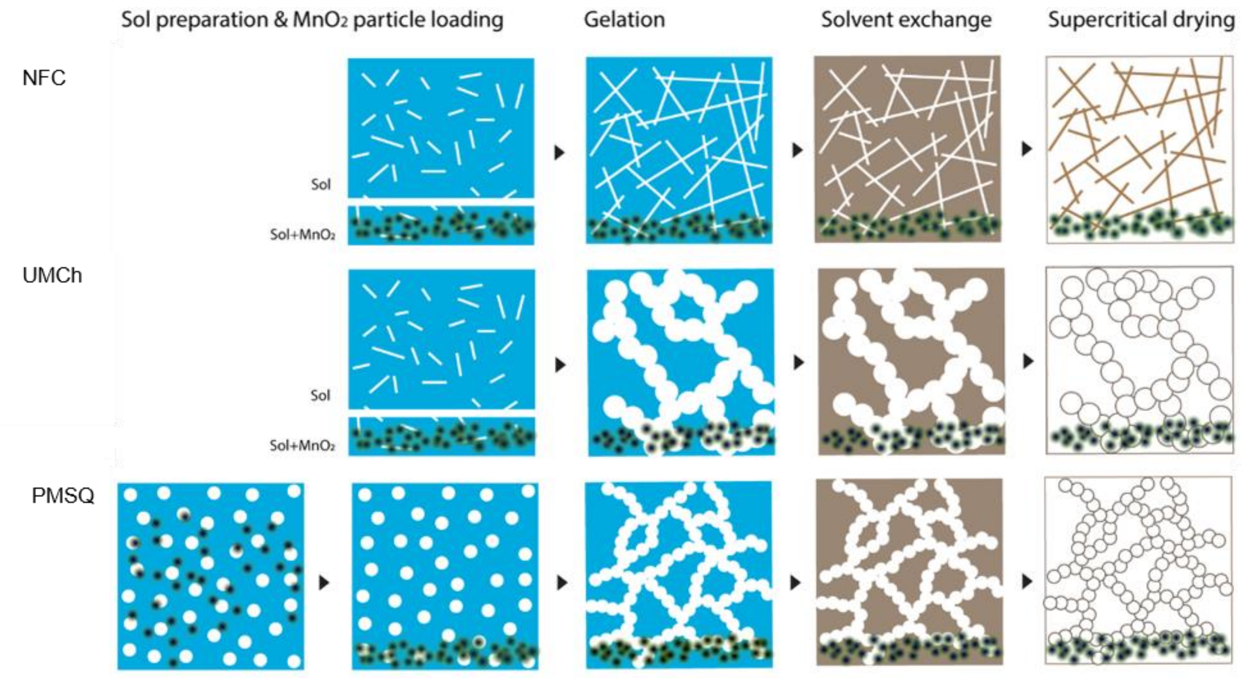

Fig. S1. Illustration of aerogel-MnO_2 composites preparation routes displaying different procedures for PMSQ, where the pure aerogel sol was casted on the bottom of MnO_2 and aerogel sol mix for NFC and UMCh membranes whereas the absorber layer was prepared by mixing of MnO_2 and PMSQ sol followed by settlement of MnO_2 particles.

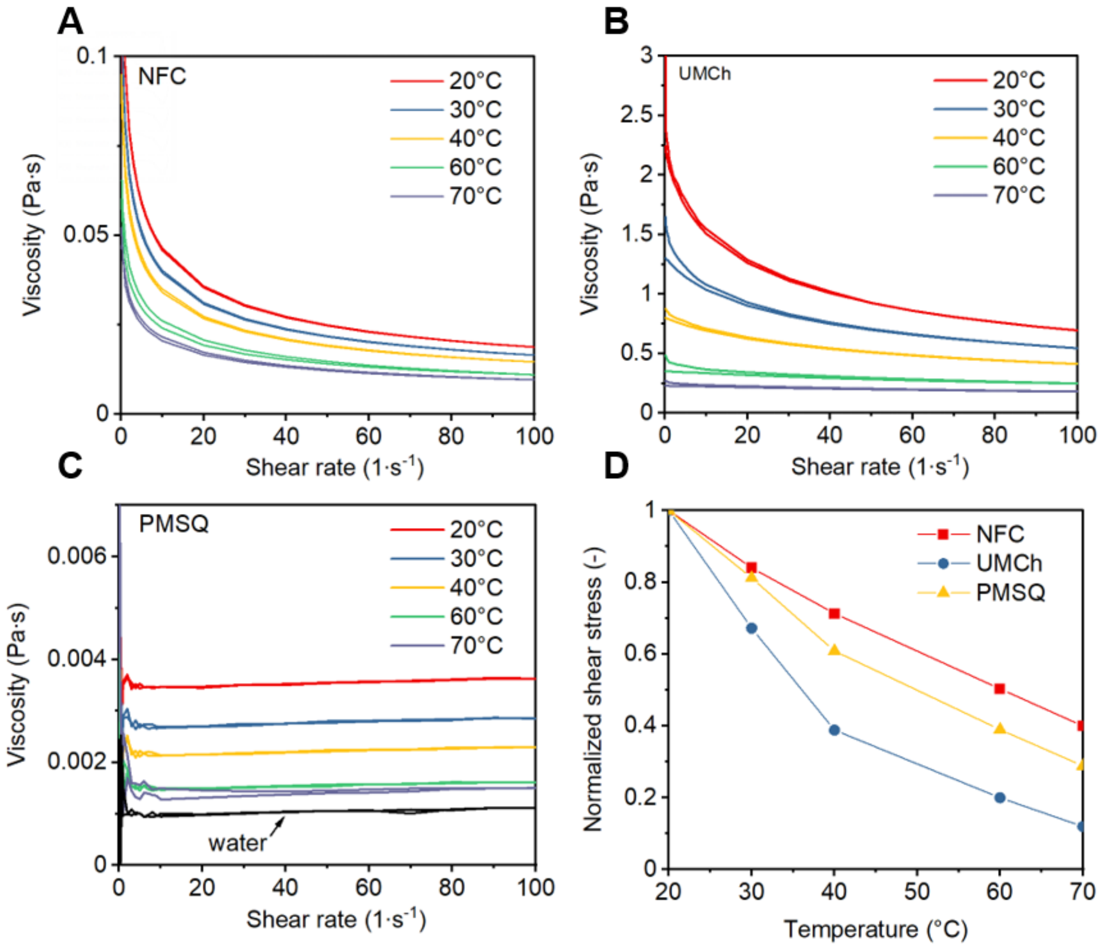

Fig. S2. Rheology analysis for (A) NFC, (B) UMCh and (C) PMSQ. (D) Normalized shear stress with respect to increased temperature. These results demonstrate the differences in the viscosity of the aerogels. It provides insight into the preparation processes aerogel samples with the absorber layer. The low viscosity and Newtonian fluid character of PMSQ sol allowed for the settling of MnO_2 particles after the mixing with PMSQ sol. On contrary, NFC and UMCh samples needed to be prepared by the casting of MnO_2-aerogel sol mixture and neat aerogel sol. UMCh sol, however, enabled the formation of much thinner layer than NFC due to the effect of high temperature during the preparation process, which changed the non-Newtonian fluid character towards Newtonian. As represented by the normalized shear stress (D), the viscosity of UMCh is sensitive to the temperature increase during the preparation procedure, which resulted in the formation of compact absorber layer, similar to the PMSQ sample.

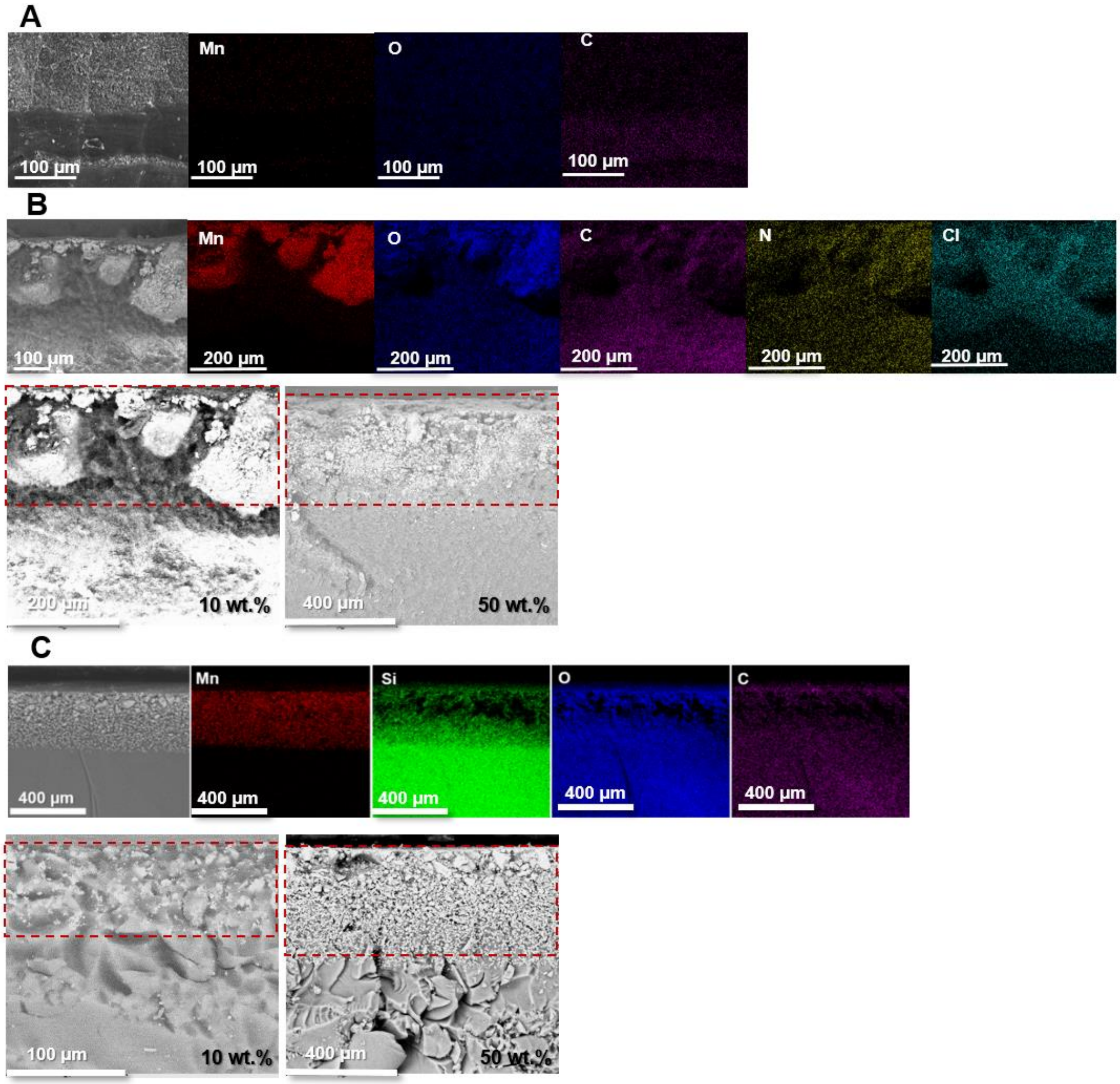

Fig. S3. SEM-EDX elemental analysis and absorber layer thickness representation for (A) NFC and (B) UMCh (C) PMSQ membrane cross-section displaying the distribution of Mn, Si, O, C, N, and Cl elements within the membrane structures. Red dashed rectangles show the thickness of the top absorber layer.

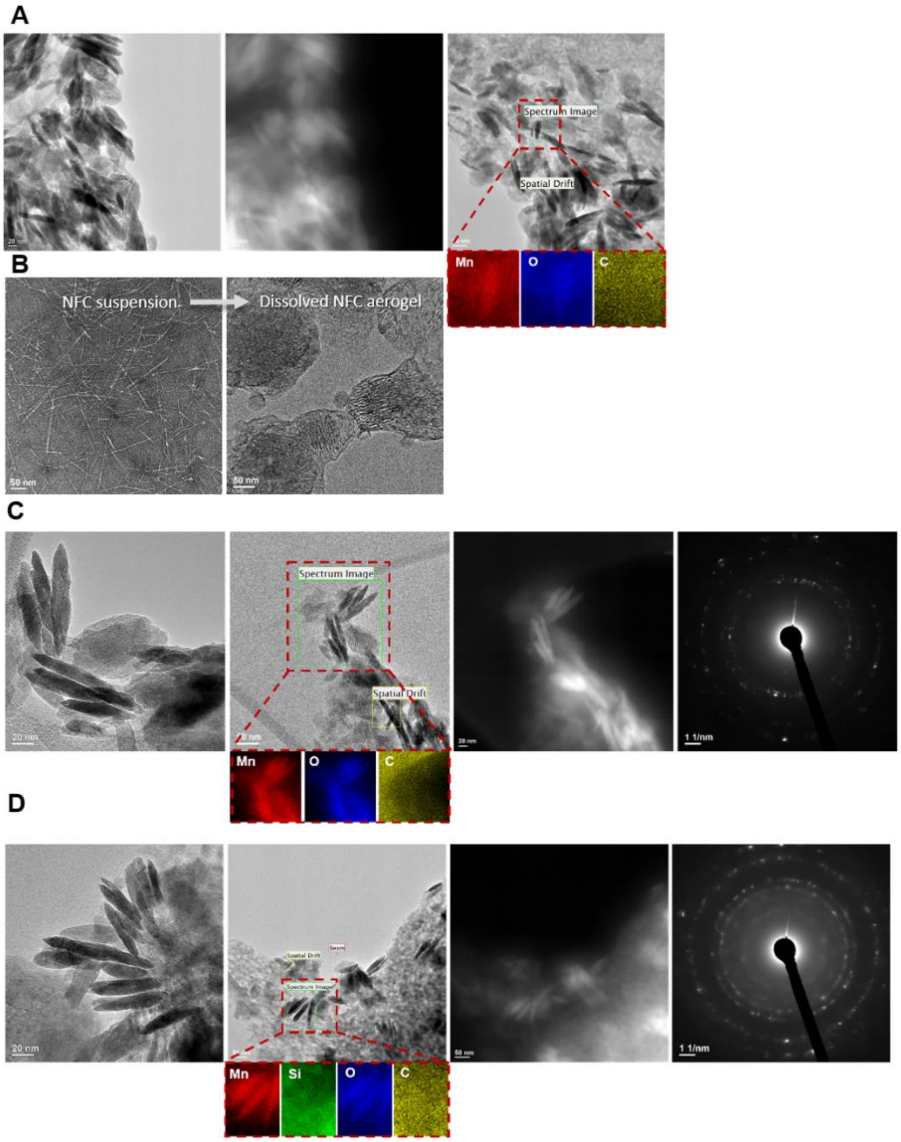

Fig. S4. TEM and STEM elemental analysis and SEAD images for (A and B) NFC, (C) UMCh and (D) PMSQ showing the compacted MnO_2 nanoflakes embedded in aerogel matrixes. Due to the TEM NFC sample preparation, the original NFC structure was dissolved, and thus, no fibre structure is visible from FC aerogel TEM images ((B) TEM of pure NFC suspension and dissolved MnO_2-NFC aerogel after TEM sample preparation).

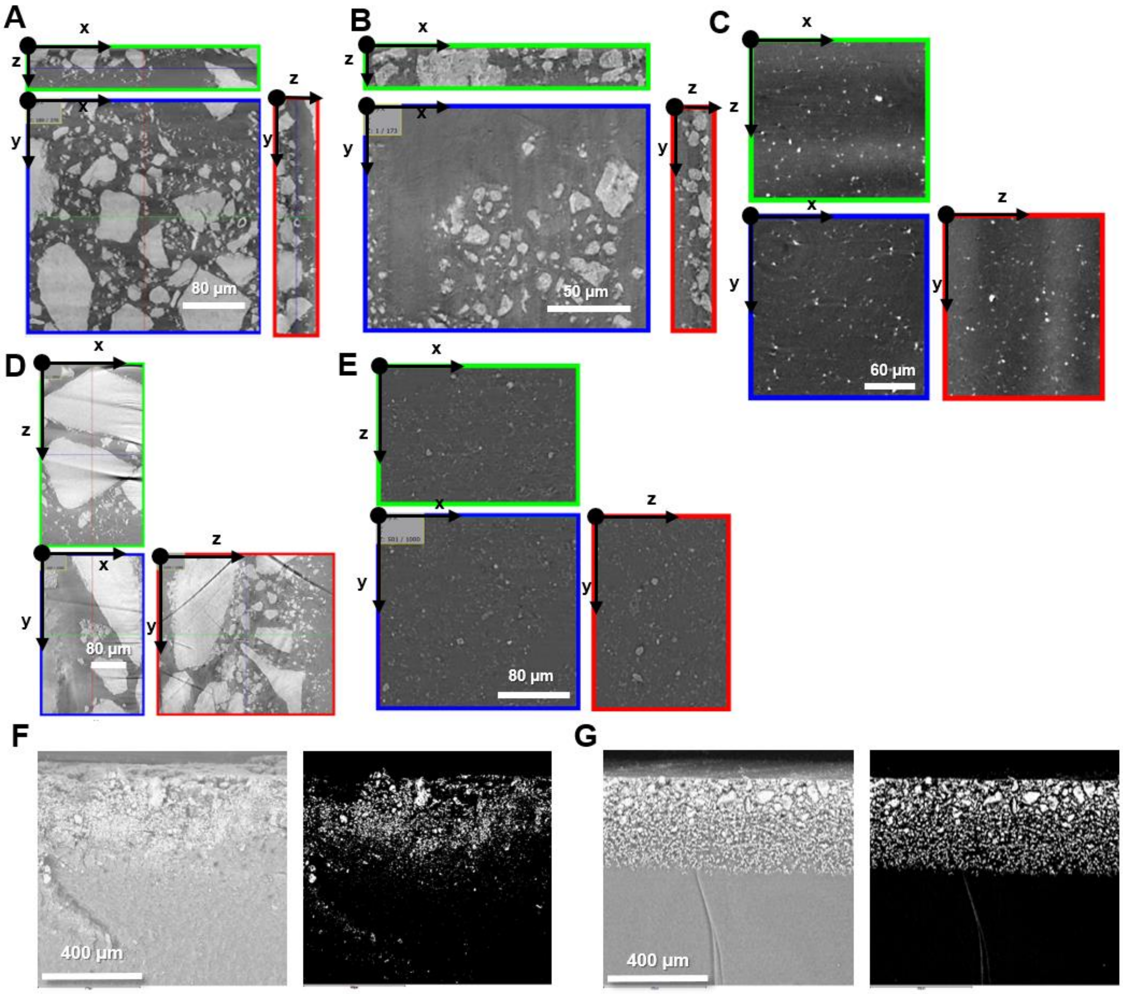

Fig. S5. Tomographic 2D images of absorber layer scanned in x, y and z dimension and SEM images of aerogel cross-section showing MnO_2 aggregate size for (A) PMSQ, (B) UMCh, (C) NFC with 10 wt.% MnO_2 loading and (D) PMSQ and (E) NFC with 50 wt% MnO_2 loading. SEM images with high contrast processing display the dominated smaller particle size for (F) UMCh sample comparing to (G) PMSQ sample both with 50 wt.% MnO_2 loading. The observation from SEM supports the observation from tomographic analysis.

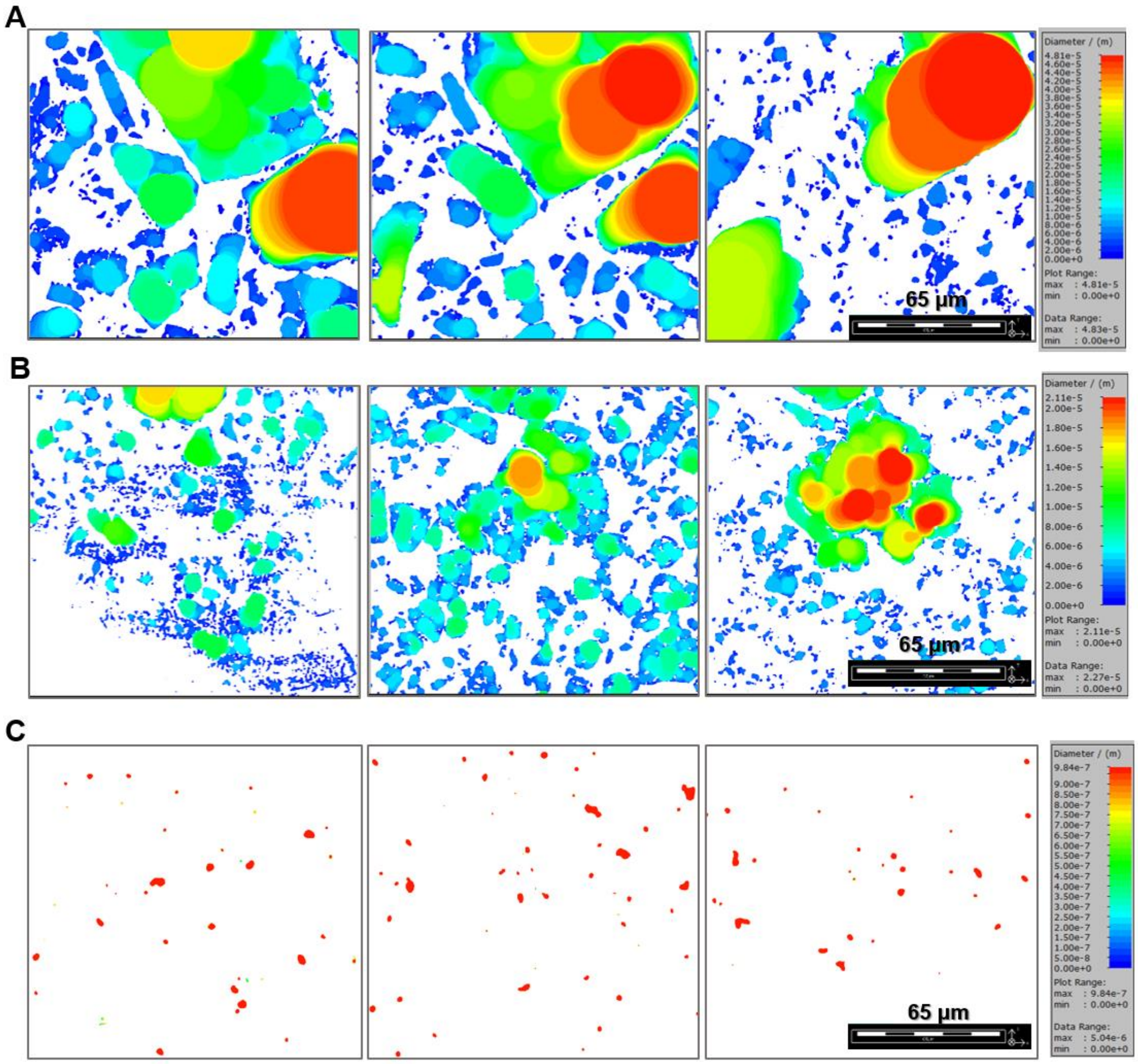

Fig. S6. Particle size analysis using tomographic images of (A) PMSQ, (B) UMCh, (C) NFC.

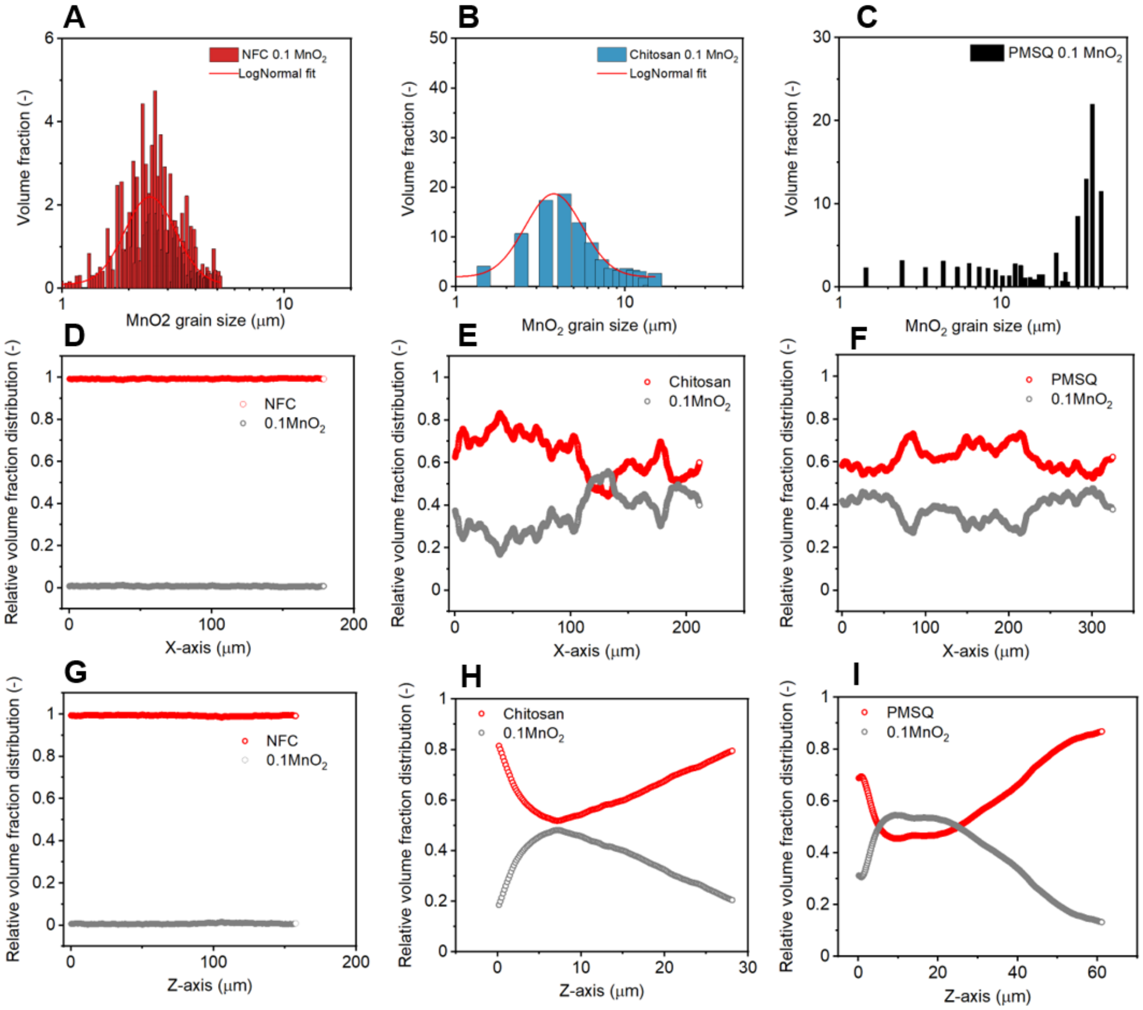

Fig. S7. Tomographic analysis of 10 wt.% MnO_2 loaded aerogels. (A-C) Agglomerate/grain size distribution for PMSQ, UMCh and NFC aerogels' top layer containing MnO_2. (D-F) Relative volume fraction distribution of MnO_2 in aerogel matrix across x-axis and (G-I) across y-axis. The averaged MnO_2 agglomerate/grain sizes were determined as 2.2 μm, 4.7 μm and 20.9 μm for NFC, UMCh and PMSQ sample.

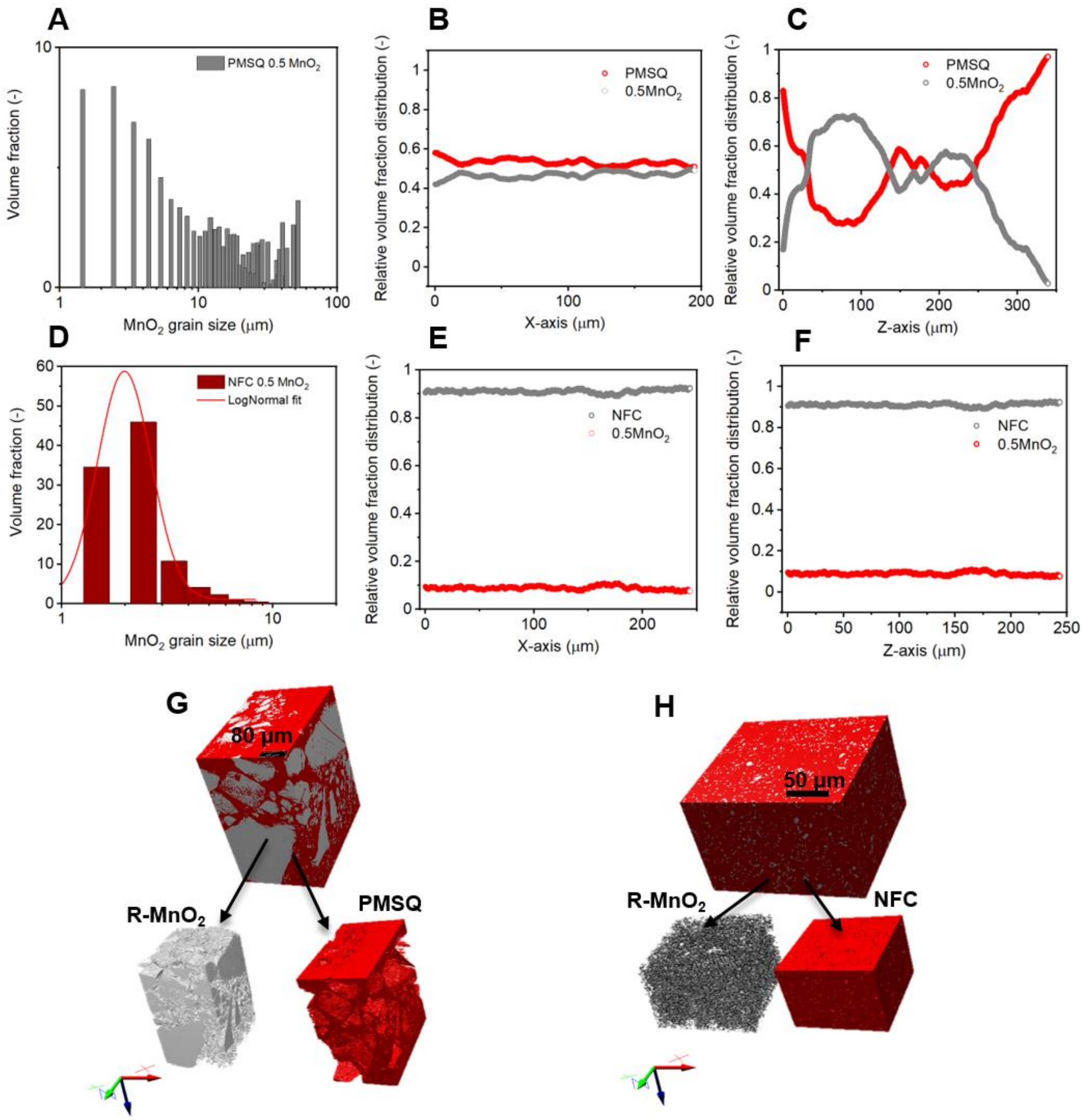

Fig. S8. Tomographic analysis of 50 wt.% MnO_2 loaded aerogels. Agglomerate/grain size volumetric distribution (A) and relative volume fraction distribution of MnO_2 in PMSQ aerogel matrix across (B) x-axis and C, z-axis. Agglomerate/grain size volumetric distribution (D) and relative volume fraction distribution of MnO_2 in NFC aerogel matrix across (E) x-axis and (F) z-axis. Tomographic 2D images of aerogels' top layers and its 3D reconstructions for G, PMSQ and (H) NFC (Red-aerogel, grey-MnO_2).

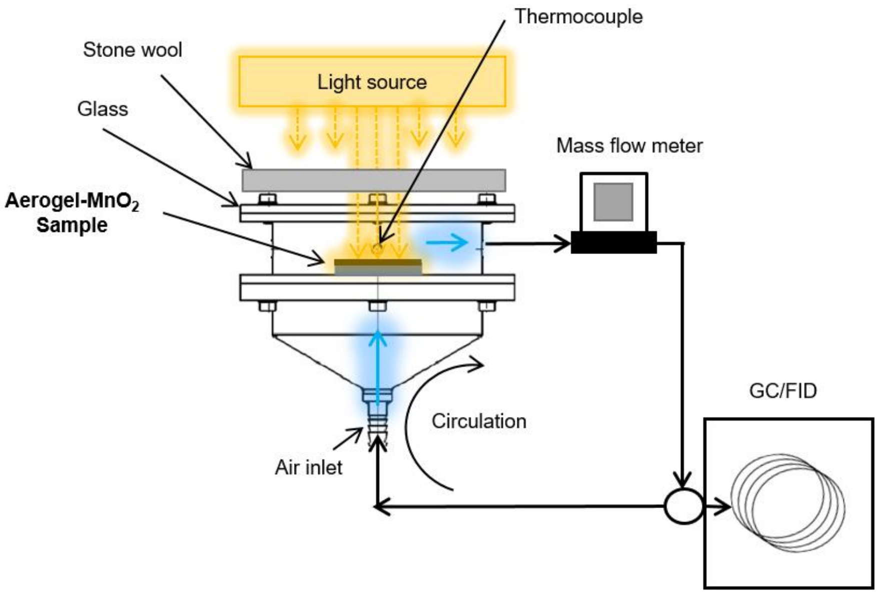

Fig. S9. Pumping and VOCs degradation testing set-up consisting of a reactor, gas chromatograph coupled with a flame ionization detector, and a light source that irradiates an aerogel-MnO_2 sample through a glass window of the reactor. Stone wool was used to insulate the heat from the lamp in order to maximize the temperature difference across the sample. Mass flow meter was used to measure the flow rate that was induced by the irradiation of aerogel-MnO_2.

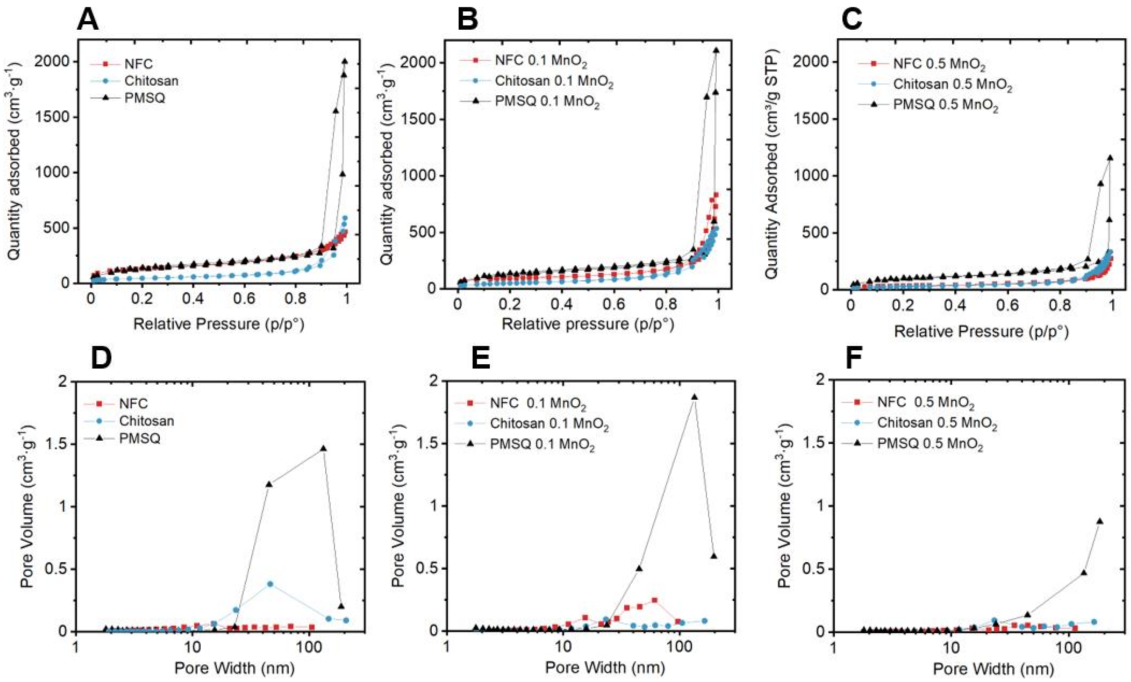

Fig. S10. N_2 sorption isotherms and pore size distribution below 300 nm according BJH adsorption model for (A and D) pristine NFC, UMCh and PMSQ, and with (B and E) 10 wt.% loading and (C and F) 50 wt.% loading of MnO_2. The size distribution is shifted towards larger pores with increasing amount of MnO_2.

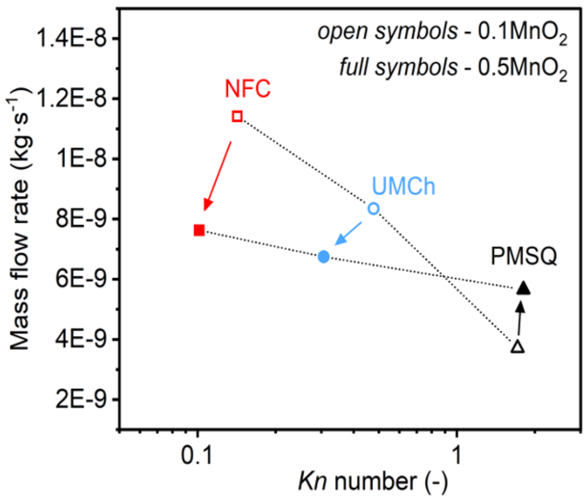

Fig. S11. Mass flow rate as a function of *Kn* number. While the increase of MnO_2 loading in PMSQ leads to the increase of the mass flow rate, it reduced the mass flow rate for NFC and UMCh samples significantly.

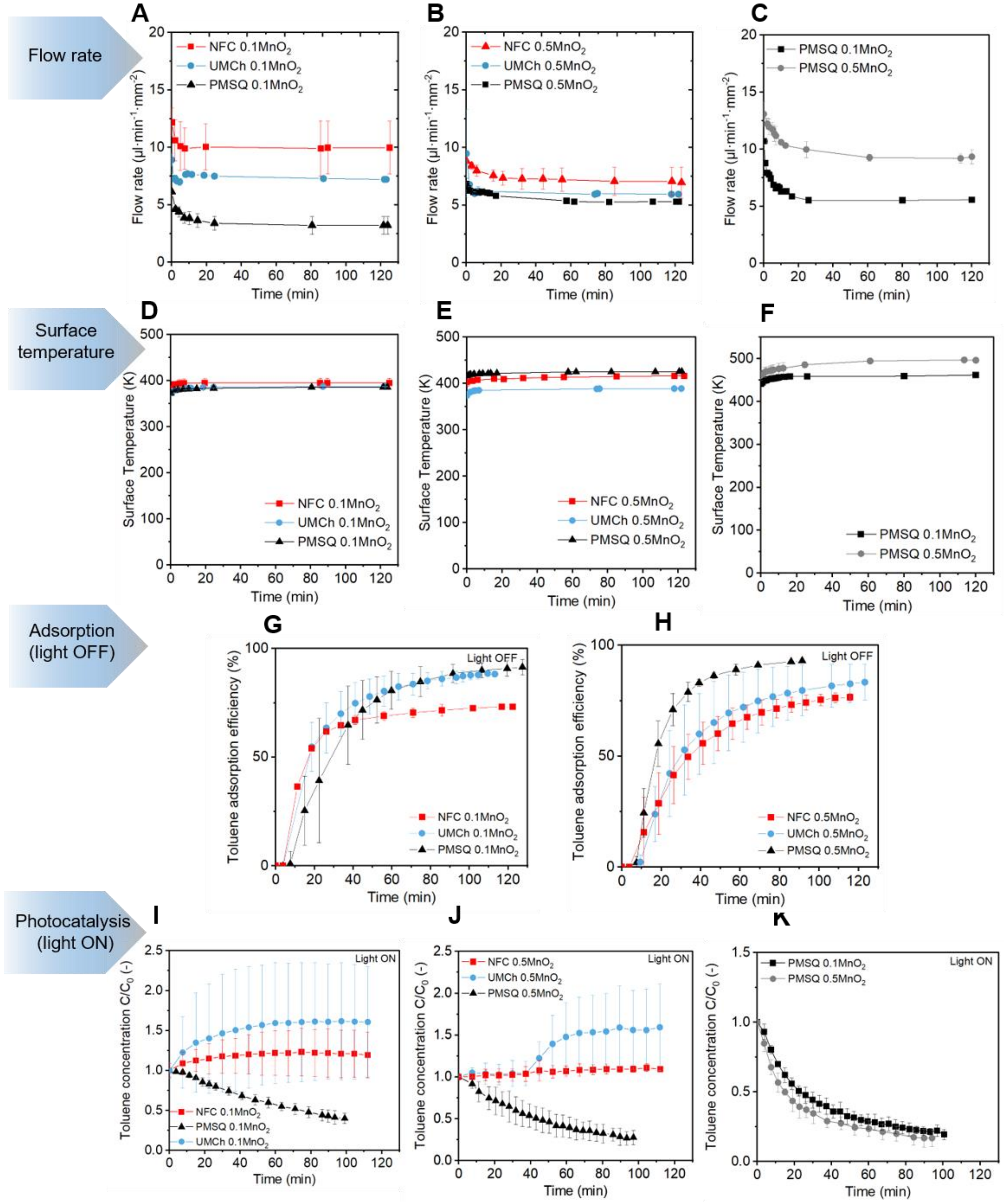

Fig. S12. Real time evolution of flow rates (A-C), surface temperatures (D-F), adsorption efficiencies (G,H) and photothermocatalytic (I-K) performances for 10 wt.% and 50 wt.% MnO_2 loading for NFC, UMCh, and PMSQ samples over around 2h irradiation with 97 $mW{\cdot}cm^{-2}$ irradiation intensity and for PMSQ with 222 $mW{\cdot}cm^{-2}$. The course of pumping performance is characterized by a higher flow rate after the sample irradiation due to thermal expansion and higher temperature differences in the membrane

and reactor. After approximately 10 minutes, the flow rate stabilizes as the temperature difference reaches a steady state. The surface temperature increases immediately after the sample is irradiated corresponding to the pumping performance with approx. 10 min stabilization.

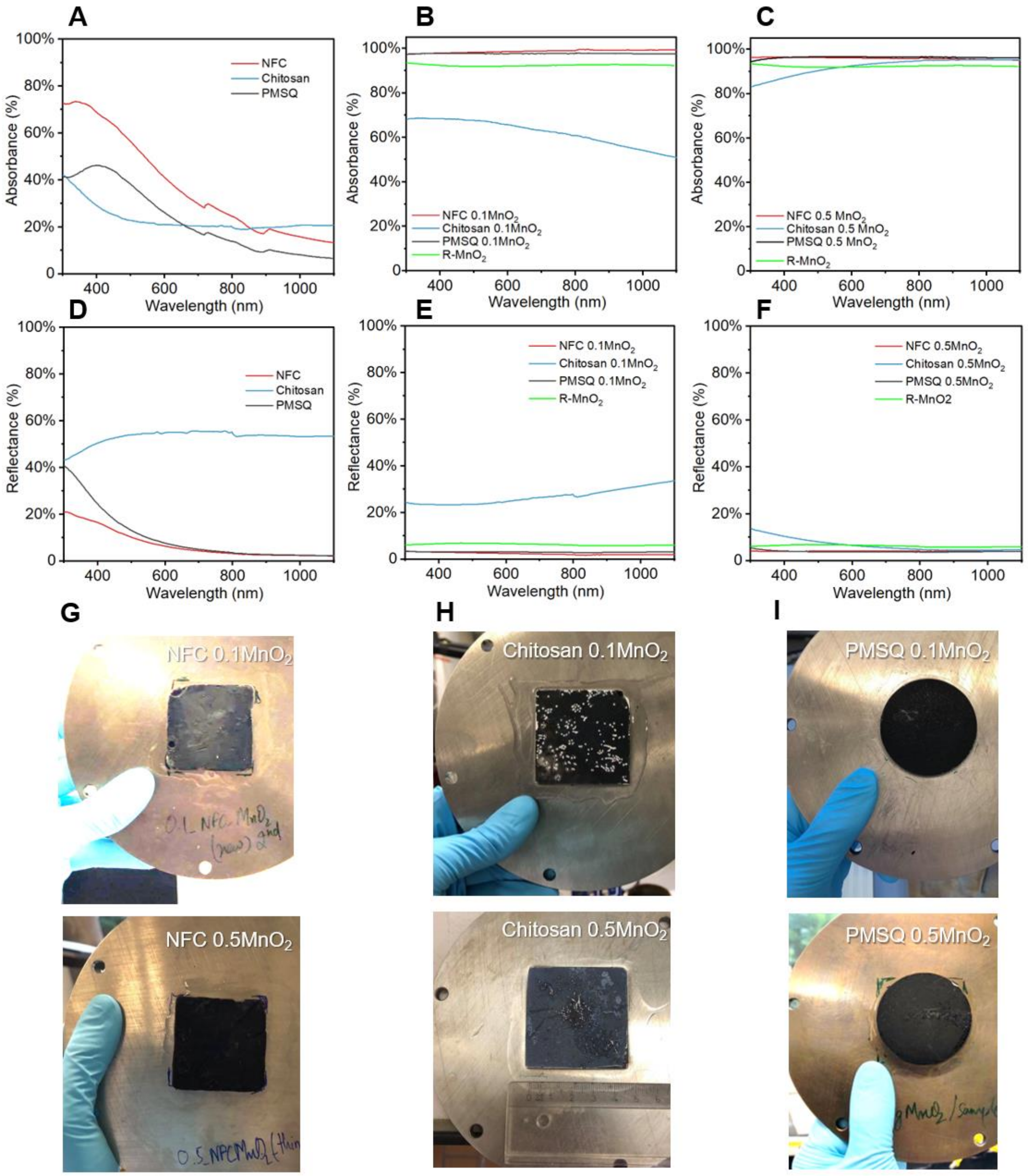

Fig. S13. UV-Vis-NIR spectrophotometry. Absorbance and reflectance spectra of (A and D) pure aerogels, (B and E) aerogels with 10 wt.% loading and (C and F) with 50 wt.% loading. (G-I) top view of aerogels' absorber layers. The results show high absorption in the whole solar spectra, expect of the UMCh with 10 wt.% R-MnO_2 loading, which can be explained by the presence of (H) white areas of pristine UMCh as a result of the sample preparation. For 50 wt.% loading, the white areas are less pronounced.

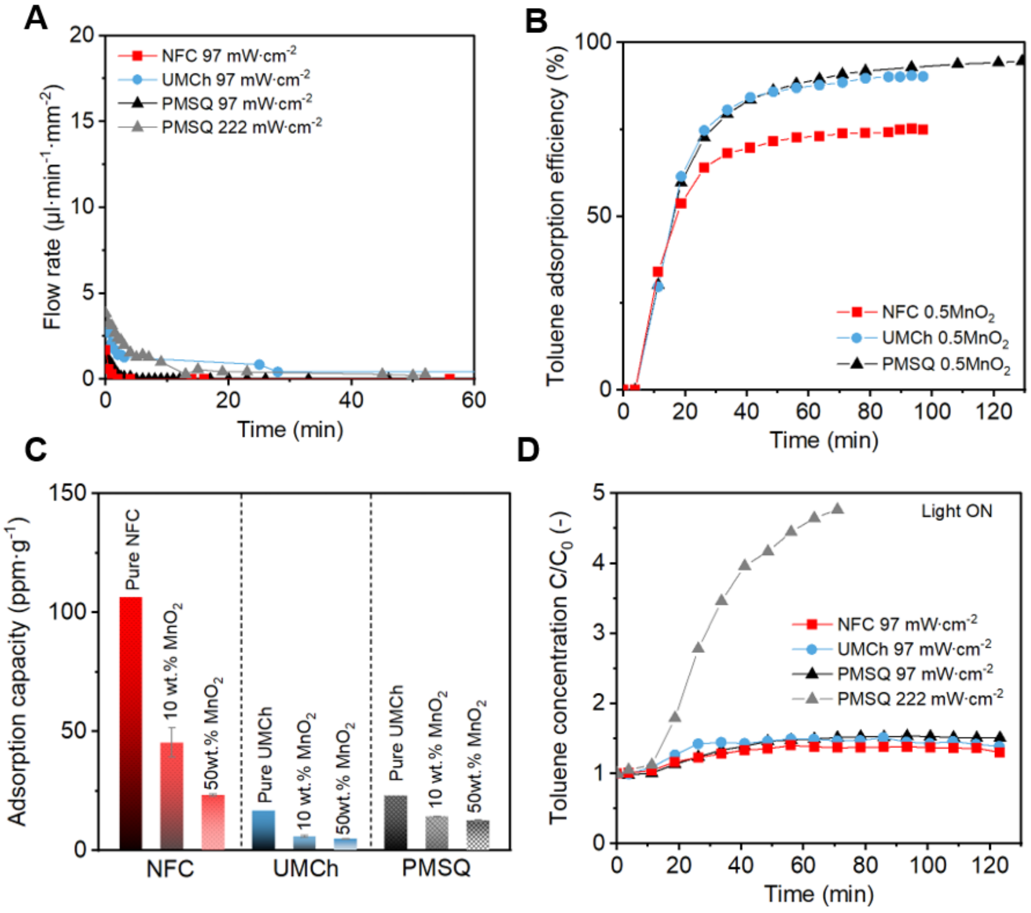

Fig. S14 A) Pumping performance, B) toluene adsorption efficiency and C) capacity, and D) toluene degradation performance of pristine aerogels without the absorber layer. Because pristine aerogels are mostly infrared transparent, the light absorption is limited and the temperature gradient needed for generating the Knudsen pumping is minimized with the time, thus, the flow rate drops to zero. This demonstrates the essential contribution of the MnO_2 absorber layer. On the other hand, the addition of MnO_2 nanoflakes reduces the adsorption capacities (normalized by the sample mass) comparing to the pure aerogels, because the specific surface area of MnO_2 nanoparticles is smaller than the aerogel. As expected, no catalytic activity is observed by pristine aerogels. The toluene concentration increases in the absence of MnO_2 absorber due to desorption generated by the elevated temperature during the irradiation.

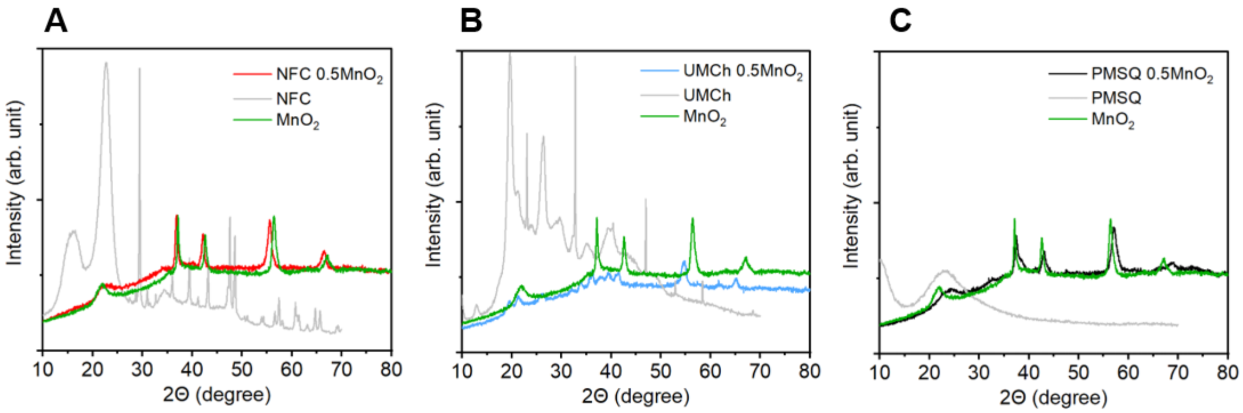

Fig. S15. Comparison of X-ray diffraction patterns of absorber layer, pristine aerogel and neat MnO_2 for (A) NFC, (B) UMCh, and (C) PMSQ. The XRD patterns show that the crystalline structures of pristine aerogels are covered and only the neat MnO_2 is visible for the composite samples. As-synthetized manganese oxide corresponds to a heterogeneous phase consisting of hexagonal Akhtenskite (ε-MnO_2) and orthorhombic ramsdellite (γ-MnO_2) (Ref. 01-089-5171 for akhtenskite and Ref. 01-073-1539 for ramsdellite) [1, 2].

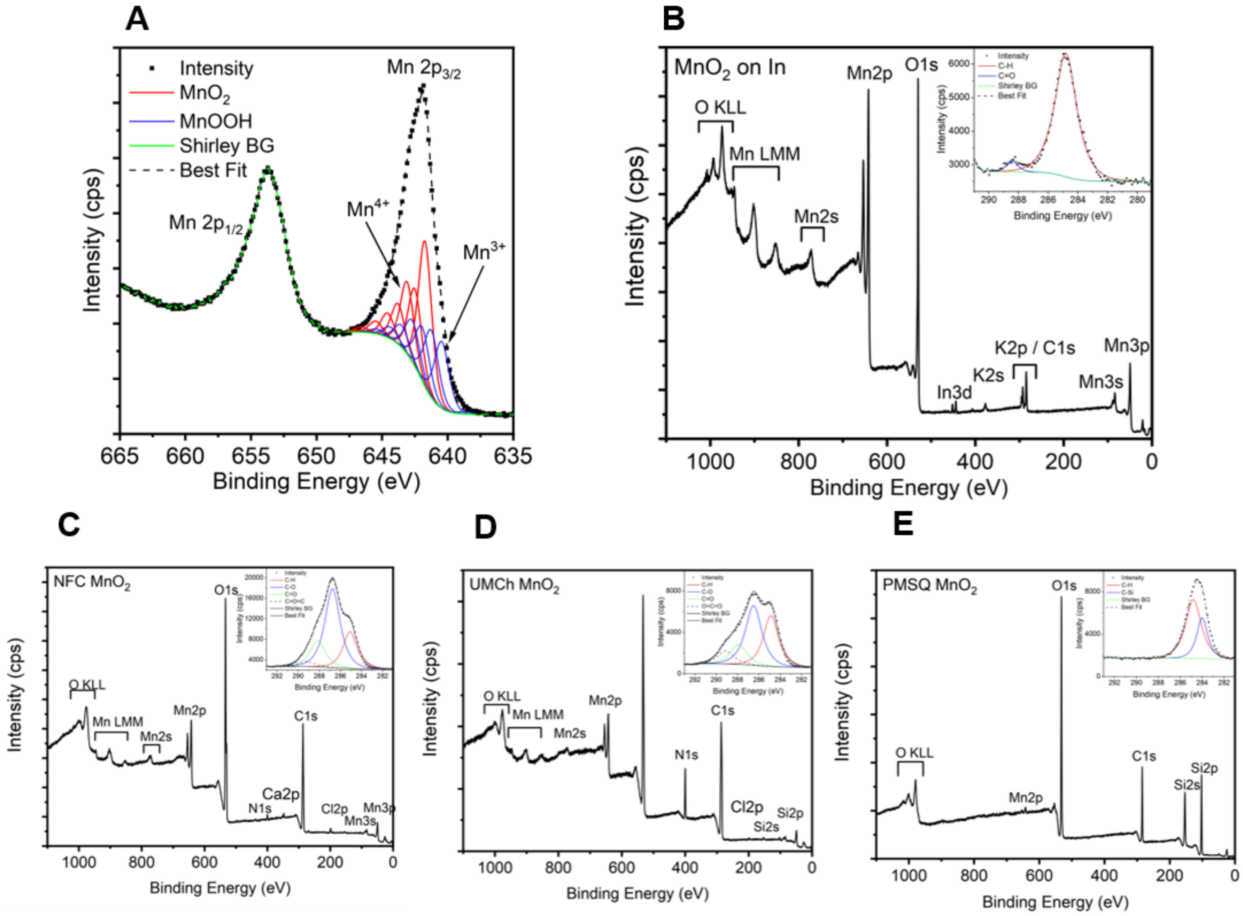

Fig. S16. (A) $Mn2p_{1/2}$ and $Mn2p_{3/2}$ electron energy level with empirical fitting of the $Mn2p_{3/2}$ peak. X-ray photoelectron spectroscopic (XPS) survey spectra of (B) neat MnO_2, (C) NFC, (D) UMCh, and (E) PMSQ samples with 50 wt.% with C 1s core level used for the calibration. Only 50 wt.% loading results are presented as they produced stronger XPS signal of MnO_2 component. The XPS analysis has been conducted to determine the effect of aerogel chemical composition of the electronic structure of MnO_2. In addition to XPS analysis, HAXPES analysis has been performed. This technique allows for the characterization of deeper lying material and reduces the effects of surface modifications and contamination. Therefore, the average oxidation state (AOS) of neat MnO_2 determined by XPS is lower (3.4+) comparing to the AOS (3.7+) measured by the HAXPES technique where the oxidation-states reducing structural defects are less prominent. Our findings further show that the surface of all samples shows significant presence of Mn (3+) species, and that the surface concentration of Mn in all composite samples is low (<5% Mn). Even though, the catalysis is possible for PMSQ sample. This indicates that the irradiation penetrate into the absorbed layer of aerogels and the limited degradation of NFC and UMCh is mainly caused by the blockage of MnO_2 surface active sites with the functional groups of NFC and UMCh matrixes.

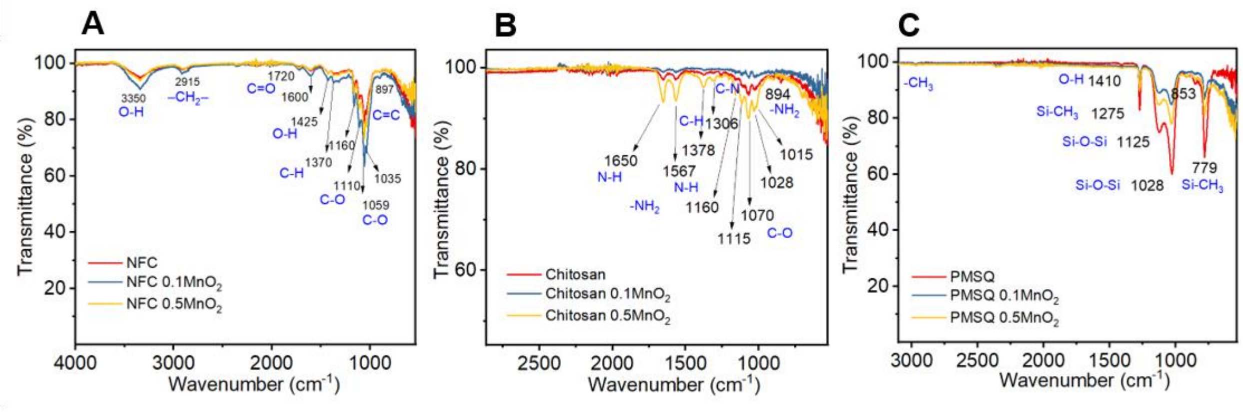

Fig. S17. Identification of surface functional groups using FTIR spectroscopy for (A) NFC, (B) UMCh, (C) PMSQ. NFC displays the main patterns in the broad band for O–H stretch @ 3300 - 2500 cm^{-1}, sharp band for C=O stretch @ 1760 - 1690 cm^{-1}, C-O stretch @ 1320 - 1210 cm^{-1}, O-H bend @ 1440 - 1395 cm^{-1} and 950 - 910 cm^{-1}. The NFC is enriched with carboxylate groups by TEMPO-mediated oxidation, which are considered as metal-binding groups [3]. UMCh shows the main sharp bands for N-H stretch @ 1650 - 1580 cm^{-1}, C-H stretch @ 1650 - 1580 cm^{-1}, C-N stretch @ 1342 - 1266 cm^{-1} and C-O stretch @ 1085 - 1030 cm^{-1}. UMCh possessed three main reactive functional groups, an amino/acetamido group, and primary and secondary hydroxyl groups. The amino groups randomly distributed providing centres for binging metals though chelation and electrostatic attractions [4]. Both NFC and UMCh surface functional groups affects the catalytic activity of MnO_2 nanoflakes by binding on its surface. PMSQ shows the main sharp bands for CH_3 deformation in Si-CH_3 stretch @ 1300 - 1200 cm^{-1} and for Si-O-Si stretch @ 1200 - 1000 cm^{-1}, with the separation of two peaks at ~1125 cm^{-1} for the Si-O-C bond and at ~1028 cm^{-1} for the Si-O-Si bond. The sharp band for -CH_3 rocking and Si-C stretching in Si-CH_3 was found @ 790 – 760 cm^{-1} [5].

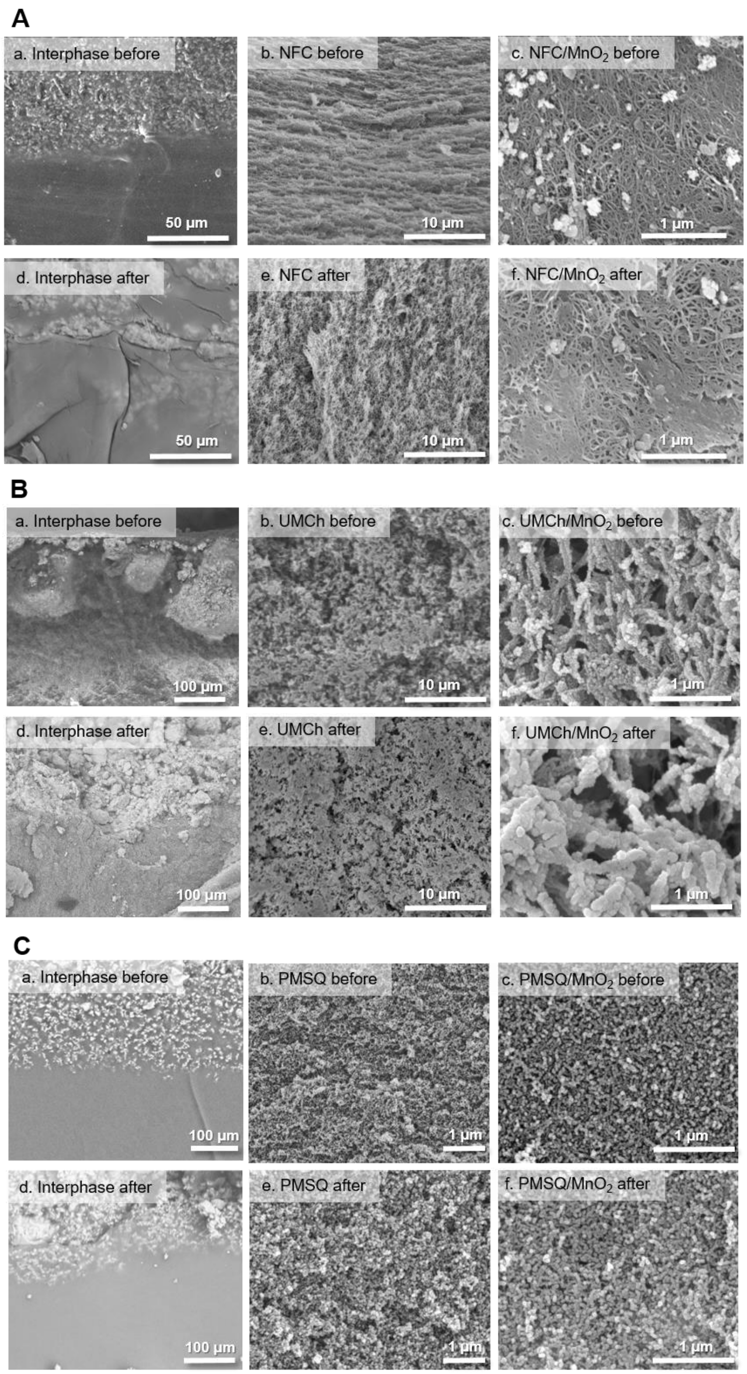

Fig. S18. SEM images of absorber layer interface, bottom aerogel layer and absorber surface layer of A) NFC, B) UMCh, and C) PMSQ samples before and after pumping, adsorption and degradation tests (including irradiation). The morphology of the interphase layers and bottom aerogel layers seem to be stable. On the other hand, the surface layers of NFC and UMCh samples display structure changes as the aerogel particles were annealed due the elevated temperature during the irradiation and the aerogel particles partially merged.

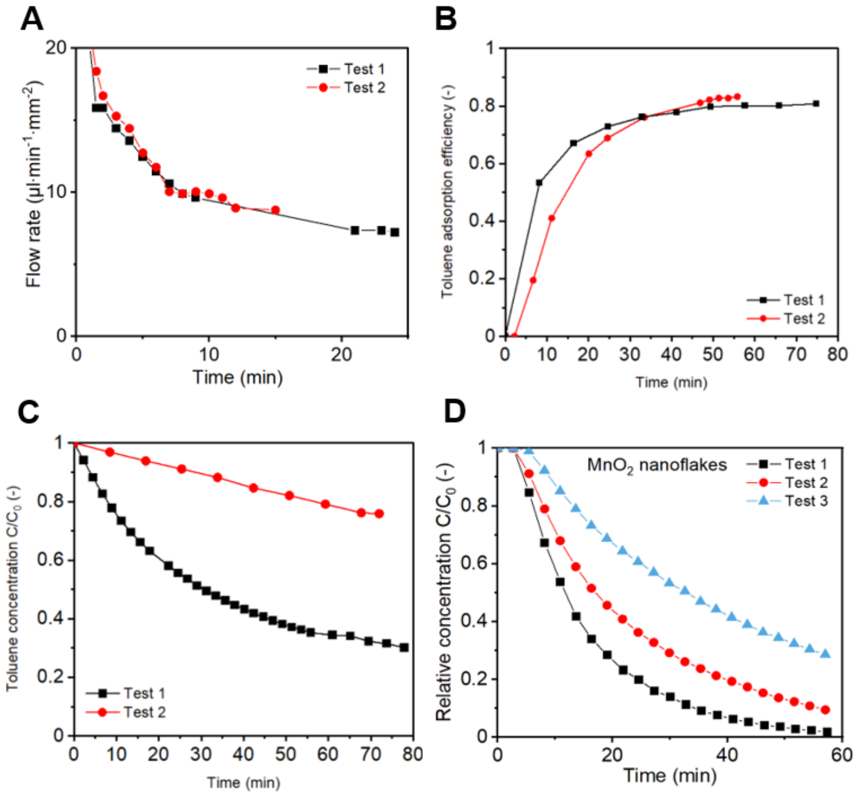

Fig. S19. Preliminary evaluation of recyclability. A) Pumping performance of silica aerogel MnO_2 bilayer samples using 222 mW·cm^{-2} irradiation intensity. B) Two subsequent adsorption tests on silica aerogel MnO_2 bilayer sample followed by C) degradation tests. D) The recyclability of pristine MnO_2 nanoflakes deposited onto glass fibre filter and irradiated with the intensity of 222 mW·cm^{-2}. The deactivation is observed for the silica aerogel membranes containing MnO_2 absorber layers as well as for the pristine MnO_2 nanoflakes. The activity decreased by 45% for silica-MnO_2 sample in the second cycle. The pristine MnO_2 was reduced by around 8% in the second cycle and by almost 25% in the third cycle. The deactivation depends on several conditions, such as the type of sample and pollutant, the reaction duration or the amount of adsorbed water [6, 7]. In general, the complete avoidance of catalyst deactivation is not possible, for example, the deactivation of almost 37% and 55% in the second and third cycle, respectively, has been reported during toluene degradation onto benchmarked TiO_2 photocatalyst [8].

Table S1. Zeta potential results, which demonstrate destabilization of the repulsive interactions of MnO_2 in the PMSQ sol in comparison to the stabilization tendency in UMCh and NFC sols. The results show slight decrease of zeta potential when MnO_2 is added to the aerogel sols indicating slight destabilization of aerogel sols. On the other hand, the stability of MnO_2 particles is influenced significantly, i.e. MnO_2 particles are stabilized by NFC and UMCh sols and destabilized by PMSQ sol.

	Zeta potential (mV)		
	PMSQ	NFC	UMCh
Sol[a]	16	-69	74
MnO_2 in solvent[b]	44	-36	27
**MnO_2 in sol**	**14**	**-68**	**72**

[a]Aerogel sol – methyltrimethoxysilane (MTMS) for PMSQ, NFC suspension, UMCh sol, [b] solvent – water and CTAC Urea for PMSQ and UMCh, water for NFC.

Table S2. State-of-art summary of micropumping techniques

Micropumping technique	Actuation method	Q_{max} (µl/min), Q_{max}/A_p (µl/min mm^2)	$p_{back,max}$ (kPa)	V (V), V/Q (V min/ml)	P (W), P/Q (W min/ml)	Planform, outlet areas (mm × mm)	Out of Plane (mm)	Frequency	Reference
**Mechanical displacement pumps**						dia 1.56, dia 1.56[a]			
Vibrating diaphragm	PVDF	106 (air), 55.5	–	106 (V/µm), 1,000 (V.min/ml µ m)	–	5 × 5, 0.078 × 0.078	0.4	100 Hz	[9]
Vibrating diaphragm	Electrostatic	1 (DI water), 0.04	–	50, 50000	–	10 × 6[a], dia 1.2	0.063	1.83 kHz	[10]
Vibrating diaphragm	Magnetic, DC motor	774 (water), 12.9	1.7 (6.2 Hz)	–	0.013, 0.017	10 × 6[a], dia 1.2	2[a]	5.9 Hz	[11]
	Magnetic, coil	1000 (water), 16.7	7.5 (4 Hz)		0.5, 0.5	dia 10, 1 × 0.4		5 Hz	
Vibrating diaphragm	Magnetic, ball valves	5000 (water), 63.7	28	–	–	6.5 × 3.5, dia 1	2.5	30 Hz	[12]
Vibrating diaphragm	Thermo-pneumatic	0.078 (methanol), 0.034	–	55, 705128	–	6.5 × 3.5, 0.08 × 0.35[a]	1	6 Hz	[13]
Vibrating diaphragm	Piezoelectric	32900 (methanol), 1446	0.173	150, 4.56	–	dia 9, 0.25 × 0.5	0.08	300 Hz	[14]
Vibrating diaphragm	Electro-magnetic	400 (water), 6.29	1.3	–	–	dia 5.5, 0.15 × 0.15	0.5	12 Hz	[15]
Vibrating diaphragm	Piezoelectric	3.2 (water), 0.135	0.12	80, 25000	0.003, 0.938	15 × 1, 1 × 0.1	1.6	60 kHz	[16]
Vibrating diaphragm	Piezoelectric with throttles	750 (water), 50	18	140 (pp), 187	–	30 × 11, 0.4 × 0.4[a]	0.1	1.6 kHz	[17]
Vibrating diaphragm	Piezoelectric	1800 (water), 5.45	60	250, 139	0.084, 0.047	17 × 14, 0.12 × 0.12	1	27.8 Hz	[18]
Vibrating diaphragm	Piezoelectric	1300 (water), 5.46	4.1	400 (pp), 308	–	12 × 6, dia 0.6	0.12	200 Hz	[19]
Vibrating diaphragm	Solid–liquid phase change	0.074 (water), 0.001	920	–	–	2 × 2, 2 × 2	2	0.5 Hz	[20]
Vibrating diaphragm	Electrostatic	0.18 (air), 0.045	7	100 (pp), 555555	–	10 × 10 1 × 0.2	0.1	14 kHz	[21]
Vibrating diaphragm	Piezoelectric	323 (water), 3.23	0.29	385, 1192	–	1.5 × 9, 0.4 × 0.4	0.2	130 Hz	[22]
Vibrating diaphragm	Pneumatic	2600 (water), 192.6	25	–	–	20 × 0.431, 0.431 × 0.045	0.19	50 Hz	[23]
Peristaltic	Pneumatic	7.5 (water and glycerol), 0.87	–	–	–	8 × 2.5, 0.1 × 0.047	0.045	250 Hz	[24]
Peristaltic	Thermo-pneumatic	21.6 (water), 1.08	3.5	20, 925.9	–	3.6 × 14, 0.5 × 0.1	550	2 Hz	[25]
Peristaltic		13 (water), 2.14	172	–	–	9 × 9, 3 × 0.3	0.1	10 Hz	[26]
Peristaltic	Magnetic Fluid	3.8, 0.06				75 × 24, 1[a] × 0.2	0.3		[27]
Peristaltic	Piezoelectric	36.8 (water),	0.52	100, 2717	0.4, 10.9	8 × 16, dia 0.8	1.1	700 Hz	[28]

		0.02							
Peristaltic (Back pressure Independent)	Piezoelectric	30 (water), 0.23	30	80, 2667	–	7 × 14 1 × 1[a]	–	4 Hz	[29]
Fluid displacement	Magnetic Fluid	30 (water), 0.3	2.5	–	–	1 × 0.94, 1 × 1	6	0.2[a] Hz	[30]
Fluid displacement	Gas Permeation	0.2 (water), 0.21	–	–	–	1 × 11, 0.3 × 0.4	0.1	–	[31]
Fluid displacement	Phase Change (Bubble)	10.13 (water), 0.92	0.49	30, 2961.5	3.4, 335.6	dia 2.38, 1.19 × 0.103	0.4	0.4 Hz	[32]
Rotary	Viscous, Single-sided	1000 (water), 224.8	0.643	–	7, 7	dia 2.38, 1.19 × 0.103 dia 3, dia 1[a]	0.103	5000 rpm	[33]
	Viscous, Double-sided	2100 (water), 472.0	1.19						
Rotary	Magnetic	8500 (water), 601.2	1.175	6, 0.71	–	4.5 × 4.5, 0.3 × 0.2	0.5	–	[34]
Rotary		9500 (water), 469	8	2.5, 0.263	0.165, 0.017	4.5 × 4.5, 0.3 × 0.2	0.4	–	[35]
**Electro- and magneto-kinetic pumps**						10 × 20, 10 × 0.05			
EHD	Injection	2700 (cryogenic HFE-7100), 13.5	0.55	180, 66.7	0.0063, 0.0023	dia 0.32, dia 0.05	0.05	DC	[36]
Electroosmotic	DC	1.7 (NaH_2PO_4 buffer), 0.01	10000	5,000, 2,940,000	0.875[a], 514.7	dia 0.1, dia 0.1	500	DC	[37]
Electroosmotic	DC	1.5 (EDTA–Tris buffer), 191.0	400	6,000, 4,000,000	0.18[a], 120	6.35 × 6.35, 6.35 × 6.35	6	DC	[38]
Electroosmotic	DC	~330 (2.5 mM $Na_2B_4O_7$), 10.4	–	9, 27.3	–	0.2 × 8.8, 0.2 × 0.025	0.09	DC	[39]
Electroosmotic	AC	2.7 (10–4 M KCl), 1.53	–	5, 1851.9	0.00036, 0.133	0.04 × 25, 0.04 × 0.03	0.025	10 kHz	[40]
Electroosmotic	Field-free	0.26, (PBS pH 7.0), 0.26	–	1 (V/mm), 3.84 (V·min/ml·mm)	–	8.65 × 12.65, 0.15 × 0.075	0.03	–	[41]
MHD	DC	1.5 (1 M buffer), 0.013	0.18	19, 12667	0.038, 25.3	2.3 × 0.3, 0.1 × 0.3	0.075	DC	[42]
Opto-electrostatic micro vortex (OEMV)	Opto-electrostatic	4.44 (water with oil and surfactant), 6.43	–	70, 15765	0.1, 22.5			1 MHz	[43]

Table S3. State-of-art summary of Knudsen pumps KP

**Micropumping technique**	**Actuation method**	**KP configuration/material**	**Power input (W)**	**Flow rate (ul/min cm^2)**	**Knudsen number *Kn* (-)**	**Reference**
Thermal transpiration	Resistive element	Micro-channel	0.08	0.033		[44]
Thermal transpiration	Resistive heating	Porous ceramics	0.08	0.0015		[45]
Thermal transpiration	Radiant element	Porous aerogel	3.4	0.59		[46]
Thermal transpiration	Thermoelectric material	Nanoporous polymer stack	0.5	280		[47]
Thermal transpiration	Resistive element	Porous ceramics	1.4	930		[48]
Thermal transpiration	Thermoelectric material	Nanoporous polymer (mixed cellulose)	8.5	1480		[49]
Thermal transpiration	Resistive element	Porous aerogel (silica)	3.6	3850	2.43	[50]
Thermal transpiration	Resistive element	Porous aerogel (silica)	0.344	900	2.04	[51]

Table S4i. Summary of characteristic values and the results of pumping and VOCs degradation performance for tested materials (Part 1).

Samples	MnO_2 loading (wt.%)	BET ($m^2 \cdot g^{-1}$)	$V_{poreBJH}$ ($cm^3 g^{-1}$)[1]	$D_{poreBJH}$ (nm)[2]	Porosity (%)	V_{pore} (cm^3)[3]	D_{pore} (nm)[3]	Kn[4] (l_m/D_{pore})
**NFC**	0	455	0.59	9.6	99	114.4 ± 0.7	580 ± 3.4	0.13
**0.1MnO_2-NFC**	10	303	1.20	30.4	99	74.5 ± 0.6	568 ± 4.4	0.14
**0.5MnO_2-NFC**	50	117	0.40	18.4	98	42.4 ± 4.8	839 ± 96	0.1
**UMCh**	0	164	0.90	25.3	91	6.7 ± 0.6	163 ± 15	0.42
**0.1MnO_2-UMCh**	10	174	0.82	19.9	92	7.3 ± 0.1	168 ± 3.2	0.48
**0.5MnO_2-UMCh**	50	102	0.52	19.2	91	6.8 ± 0.6	267 ± 22	0.31
**PMSQ**	0	452	3.10	32.2	92	5.5 ± 0.1	48.9 ± 0.6	1.51 (1.54)
**0.1MnO_2-PMSQ (5cm)***	10	444	3.20	35.2	91	5.2 ± 0.2	46.7 ± 1.5	1.71 (1.92)
**0.5MnO_2-PMSQ (5cm)***	50	333	1.80	26.8	89	4.0 ± 0.1	47.5 ± 1.7	1.81 (1.98)

**Values in brackets correspond to results from 5 cm irradiation distance (222 mW·cm^{-2}).* [1]BJH adsorption cumulative pore volume, [2]BJH adsorption pore diameter, [3]Calculated from density ρ and S_{BET} equation 1 and 2 (Methodology), [4]Ratio of mean free path of air molecules (equation 5) and diameter of pores D_{pore},

Table S4ii. Summary of characteristic values and the results of pumping and VOCs degradation performance for tested materials (Part 2).

Samples	XRD grain size (nm)	Q_V (µl·min^{-1}·mm^{-2})[5]	Q_M· 10^{-8} (kg·s^{-1})[6]	k (min^{-1})[7]	E (%)[8]	λ (mW·m^{-1}·K^{-1})[10]
**NFC**	–	–	-	–	–	35.7 ± 2.0
**0.1MnO$_2$-NFC**	7.2	10.0 ± 2.3	1.14	0.0006 ± 0.0002	3 ± 0	–
**0.5MnO$_2$-NFC**	7.5	7.0 ± 1.4	0.76	0.0003 ± 0.0004	3 ± 4	–
**UMCh**	–	0.6	-	–	–	27.9 ± 2.5
**0.1MnO$_2$-UMCh**	11.1	7.2 ± 0.2	0.83	0.0002 ± 0.0003	1 ± 1	–
**0.5MnO$_2$-UMCh**	11.0	5.9 ± 0	0.67	–	–	–
**PMSQ**	–	–	-	–	–	18.5 ± 2.0
**0.1MnO$_2$-PMSQ (5cm)***	7.5	3.2 ± 0.8 (5.6 ± 0.9)	0.37 (0.57)	0.009 ± 0.002 (0.024 ± 0.001)	60 ± 7 (78 ± 2)	–
**0.5MnO$_2$-PMSQ (5cm)***	5.9	5.2 ± 0.1 (9.3 ± 0.5)	0.57 (0.91)	0.016 ± 0.004 (0.037 ± 0.010)	72 ± 8 (83 ± 5)	–

[5]Volumetric flow rate, [6]Mass flow rate, [7]Degradation rate constant determined from the linear regression of $\ln(C_0/C)$ (Pseudo 1st order kinetic model), [8]Toluene degradation efficiency, [9]Average temperature difference ΔT per mm of sample thickness *t*, [10]Thermal conductivity.

Table S4iii. Summary of characteristic values and the results of pumping and VOCs degradation performance for tested materials (Part 3).

**Samples**	**T_{HOT} (K)[11]**	**T_{COLD} (K)[12]**	**ΔT (K)[13]**	**t (mm)[14]**	**ΔT/t (K·mm^{-1})[15]**
**NFC**	395	333	63	8.8	7
**0.1MnO_2-NFC**	416	350	66	10.4	6
**0.5MnO_2-NFC**	387	328	59	6.5	9
**UMCh**	389	341	49	7.0	7
**0.1MnO_2-UMCh**	386	333	53	5.0	11
**0.5MnO_2-UMCh**	426	339	87	4.7	19
**PMSQ**	395	333	63	8.8	7
**0.1MnO_2-PMSQ**	416	350	66		6
**(5cm)***	(457)	(351)	(106)	10.4	(22)
**0.5MnO_2-PMSQ**	387	328	59		9
**(5cm)***	(497)	(344)	(152)	6.5	(32)

[11]Temperature of irradiated side, [12]Temperature of cold side (pristine aerogel side), [13]Temperature difference between irradiated and cold sides, [14]Thickness of sample, [15]Temperature difference normalized by the thickness of samples.

Table S5. State-of-art summary of VOCs degradation performances using MnO_2 based catalyst activated

Photocatalyst	Catalyst structure	Coating method/Catalyst loading	Reactor type and volume	Pollutant Conc.	Residence time/flow rate	Light primary wavelength and irradiation ($W \cdot cm^{-2}$)	Reaction time (min)	Conversion (%)	1st oder rate constant (min^{-1})	Reference

by light

R-MnO_2-HS	Nanosheets	6.25 mg·cm^{-2}	Continuous, cylindrical stainless steel gas-phase reactor with a quartz window, 447 mL	Benzene, 8.0 uL	20 mL·min^{-1}	Xe lamp, 0.370	30	86.30%		[52]
CeO_2/MnO_2 120C	Nanorods/ nanocrystal s	6.25 mg·cm^{-2}	Continuous, cylindrical stainless steel gas-phase reactor with a quartz window, 447 mL	Benzene, 2.0 uL		Xe lamp, 0.3017	20	100%		
CeO_2/MnO_2 180C	Nanorods/ nanocrystal s	6.25 mg·cm^{-2}	Continuous, cylindrical stainless steel gas-phase reactor with a quartz window, 447 mL	Benzene, 2.0 uL		Xe lamp, 0.3017	20	60%		[53]
OMS-MnO_2	Nanorods	6.25 mg·cm^{-2}	Continuous, cylindrical stainless steel gas-phase reactor with a quartz window, 447 mL	Benzene, 2.0 uL		Xe lamp, 0.3017	20	48%		
B-MnO_2 nanowires	Nanowires	0.025 mmol/250mL	Three-neck glass reactor (250 ml)	Alizarin yellow R		mercury lamp, GYZ220–230V 250W	120	98%	0.038	[54]
MnO_2		100 mg	Stoppered flask (200 ml)	Malachite Green MG, 100ppm		sun light, 0.136	80	82%	0.0311 2	[55]
MnO_2				4 ml of acid Orange II (OII, 10 mg/L)		PLS-SXE300U V Xe lamp, 0.150		37%		[56]
MnO_2/TiO_2	Nanotubes			4 ml of acid Orange II (OII, 10 mg/L)		PLS-SXE300U V Xe lamp, 0.151		98%		
MnO_2/TiO_2	Nanotubes		Stainless steel reactor,35mL	Toluene, 200ppmv	5.7 mL·min^{-1}	25 LEDs, 0.0145	60	43%	0.0095 7	[57]
R-MnO_2	Nanosheets		Stainless steel, 800 mL	Toluene, 200ppmv	50 mL·min^{-1}	Halogen lamp, 0.34	375	88%		[51]
MnO_2/Mn_3O_4	Nanocubes	0.04 g		Toluene, 300ppm	60 mL·min^{-1}	Xe lamp, 0.764	60	90%		[58]
α-MnO_2	Nanocubes	0.04 g		Toluene, 300ppm	60 mL·min^{-1}	Xe lamp, 0.764	60	71%		
$LaTi_{1-x}Mn_xO_3^{+\delta}$		0.1 mg·cm^{-2}		Toluene, 200ppmv	50 mL·min^{-1}	Xe lamp, 0.65	100	51%		[59]
A-$LaTi_{0.6}Mn_{0.4}O_3^{+\delta}$		0.1 mg·cm^{-2}		Toluene, 200ppmv	50 mL·min^{-1}	Xe lamp, 0.65	100	96%		
MnO_2/C	Micro-flower	0.3 g		1272000ppm Toluene	30 mL·min^{-1}		70	87%		[60]

References and Notes

1. C. Wu, W. Xie, M. Zhang, L. Bai, J. Yang, and Y. Xie, *Chemistry – A European Journal*, **2009**. 15(2): p. 492-500 DOI: https://doi.org/10.1002/chem.200801814.
2. J. Yuan, K. Laubernds, Q. Zhang, and S. L. Suib, *Journal of the American Chemical Society*, **2003**. 125(17): p. 4966-4967 DOI: 10.1021/ja0294459.
3. T. Keplinger, X. Wang, and I. Burgert, *Journal of Materials Chemistry A*, **2019**. 7(7): p. 2981-2992 DOI: https://doi.org/10.1039/C8TA10711D.
4. J. Nie, Z. Wang, and Q. Hu, *Scientific Reports*, **2016**. 6(1): p. 36005 DOI: 10.1038/srep36005.
5. F. He, L. Zhou, M. Fang, C. Sui, W. Li, L. Yang, M. Li, and X. He, *Materials & Design*, **2019**. 173: p. 107777 DOI: https://doi.org/10.1016/j.matdes.2019.107777.
6. K. W. Shah and W. Li, *Nanomaterials (Basel)*, **2019**. 9(6) DOI: 10.3390/nano9060910.
7. P. Wu, X. Jin, Y. Qiu, and D. Ye, *Environmental Science & Technology*, **2021**. 55(8): p. 4268-4286 DOI: 10.1021/acs.est.0c08179.
8. S. Weon, J. Kim, and W. Choi, *Applied Catalysis B: Environmental*, **2018**. 220: p. 1-8.
9. T.-B. Xu and J. Su, *Sensors and Actuators A: Physical*, **2005**. 121(1): p. 267-274 DOI: https://doi.org/10.1016/j.sna.2005.01.020.
10. A. Machauf, Y. Nemirovsky, and U. Dinnar, *Journal of Micromechanics and Microengineering*, **2005**. 15(12): p. 2309 DOI: 10.1088/0960-1317/15/12/013.
11. T. Pan, S. J. McDonald, E. M. Kai, and B. Ziaie, *Journal of micromechanics and microengineering*, **2005**. 15(5): p. 1021 DOI: https://doi.org/10.1088/0960-1317/15/5/018.
12. C. Yamahata, F. Lacharme, Y. Burri, and M. A. Gijs, *Sensors and Actuators B: Chemical*, **2005**. 110(1): p. 1-7 DOI: https://doi.org/10.1016/j.snb.2005.01.005.
13. J.-H. Kim, K.-H. Na, C. Kang, and Y.-S. Kim, *Sensors and Actuators A: Physical*, **2005**. 120(2): p. 365-369 DOI: https://doi.org/10.1016/j.sna.2004.12.024.
14. Y. Kim, J. Kim, K. Na, and K. Rhee, *Proceedings of the Institution of Mechanical Engineers, Part C: Journal of Mechanical Engineering Science*, **2005**. 219(10): p. 1139-1145 DOI: https://doi.org/10.1243/095440605X31887.
15. C. Yamahata, C. Lotto, E. Al-Assaf, and M. Gijs, *Microfluidics and Nanofluidics*, **2005**. 1(3): p. 197-207 DOI: https://doi.org/10.1007/s10404-004-0007-6.
16. F. Guo-Hua and K. Eun Sok, *Journal of Microelectromechanical Systems*, **2005**. 14(2): p. 192-199 DOI: 10.1109/JMEMS.2004.839331.
17. M. C. Tracey, I. D. Johnston, J. B. Davis, and C. K. L. Tan, *Journal of Micromechanics and Microengineering*, **2006**. 16(8): p. 1444-1452 DOI: 10.1088/0960-1317/16/8/002.
18. A. Doll, M. Heinrichs, F. Goldschmidtboeing, H. J. Schrag, U. T. Hopt, and P. Woias, *Sensors and Actuators A: Physical*, **2006**. 130-131: p. 445-453 DOI: https://doi.org/10.1016/j.sna.2005.10.018.
19. T.-T. Nguyen, N. S. Goo, Y. S. Yoon, and K. J. Yoon. *A novel lightweight piezo-composite actuator micropump*. 2006.
20. R. Bodén, M. Lehto, U. Simu, G. Thornell, K. Hjort, and J.-Å. Schweitz, *Sensors and Actuators A: Physical*, **2006**. 127(1): p. 88-93 DOI: https://doi.org/10.1016/j.sna.2005.11.068.

21. A. A. Astle, H. S. Kim, L. P. Bernal, K. Najafi, and P. D. Washabaugh, *Sensors and Actuators A: Physical*, **2007**. 134(1): p. 245-256 DOI: https://doi.org/10.1016/j.sna.2006.06.027.
22. J. S. Yoon, J. W. Choi, I. H. Lee, and M. S. Kim, *Sensors and Actuators A: Physical*, **2007**. 135(2): p. 833-838 DOI: https://doi.org/10.1016/j.sna.2006.08.017.
23. W. Inman, K. Domansky, J. Serdy, B. Owens, D. Trumper, and L. G. Griffith, *Journal of Micromechanics and Microengineering*, **2007**. 17(5): p. 891-899 DOI: 10.1088/0960-1317/17/5/007.
24. J. Goulpeau, D. Trouchet, A. Ajdari, and P. Tabeling, *Journal of Applied Physics*, **2005**. 98(4): p. 044914 DOI: 10.1063/1.1947893.
25. O. C. Jeong, S. W. Park, S. S. Yang, and J. J. Pak, *Sensors and Actuators A: Physical*, **2005**. 123-124: p. 453-458 DOI: https://doi.org/10.1016/j.sna.2005.01.035.
26. C.-W. Huang, S.-B. Huang, and G.-B. Lee, *Journal of Micromechanics and Microengineering*, **2006**. 16(11): p. 2265-2272 DOI: 10.1088/0960-1317/16/11/003.
27. E.-G. Kim, J.-g. Oh, and B. Choi, *Sensors and Actuators A: Physical*, **2006**. 128(1): p. 43-51 DOI: https://doi.org/10.1016/j.sna.2006.01.021.
28. L.-S. Jang, Y.-J. Li, S.-J. Lin, Y.-C. Hsu, W.-S. Yao, M.-C. Tsai, and C.-C. Hou, *Biomedical Microdevices*, **2007**. 9(2): p. 185-194 DOI: 10.1007/s10544-006-9020-8.
29. A. Geipel, A. Doll, P. Jantscheff, N. Esser, U. Massing, P. Woias, and F. Goldschmidtboeing, *Journal of Micromechanics and Microengineering*, **2007**. 17(5): p. 949-959 DOI: 10.1088/0960-1317/17/5/015.
30. C. Yamahata, M. Chastellain, V. K. Parashar, A. Petri, H. Hofmann, and M. A. M. Gijs, *Journal of Microelectromechanical Systems*, **2005**. 14(1): p. 96-102 DOI: 10.1109/JMEMS.2004.839007.
31. M. A. Eddings and B. K. Gale, *Journal of Micromechanics and Microengineering*, **2006**. 16(11): p. 2396-2402 DOI: 10.1088/0960-1317/16/11/021.
32. J.-Y. Jung and H.-Y. Kwak, *Microfluidics and Nanofluidics*, **2007**. 3(2): p. 161-169 DOI: 10.1007/s10404-006-0116-5.
33. D. Blanchard, P. Ligrani, and B. Gale, *Sensors and Actuators A: Physical*, **2005**. 122(1): p. 149-158 DOI: https://doi.org/10.1016/j.sna.2005.03.072.
34. M. Matteucci, F. Pérennès, B. Marmiroli, P. Miotti, L. Vaccari, A. Gosparini, A. Turchet, and E. Di Fabrizio, *Microelectronic Engineering*, **2006**. 83(4): p. 1288-1290 DOI: https://doi.org/10.1016/j.mee.2006.01.259.
35. K. F. Lei, W. C. Law, Y.-K. Suen, W. J. Li, Y. Yam, H. P. Ho, and S.-K. Kong, *Proceedings of the Institution of Mechanical Engineers, Part H: Journal of Engineering in Medicine*, **2007**. 221(2): p. 129-141 DOI: 10.1243/09544119jeim189.
36. J. Darabi and W. Haixia, *Journal of Microelectromechanical Systems*, **2005**. 14(4): p. 747-755 DOI: 10.1109/JMEMS.2005.845413.
37. L. Chen, H. Wang, J. Ma, C. Wang, and Y. Guan, *Sensors and Actuators B: Chemical*, **2005**. 104(1): p. 117-123 DOI: https://doi.org/10.1016/j.snb.2004.05.013.
38. Z. Chen, P. Wang, and H.-C. Chang, *Analytical and Bioanalytical Chemistry*, **2005**. 382(3): p. 817-824 DOI: 10.1007/s00216-005-3130-7.
39. S. K. Vajandar, D. Xu, D. A. Markov, J. P. Wikswo, W. Hofmeister, and D. Li, *Nanotechnology*, **2007**. 18(27): p. 275705 DOI: 10.1088/0957-4484/18/27/275705.
40. T. S. Hansen, K. West, O. Hassager, and N. B. Larsen, *Journal of Micromechanics and Microengineering*, **2007**. 17(5): p. 860-866 DOI: 10.1088/0960-1317/17/5/003.

41. S. Joo, T. D. Chung, and H. C. Kim, *Sensors and Actuators B: Chemical*, **2007**. 123(2): p. 1161-1168 DOI: https://doi.org/10.1016/j.snb.2006.10.069.
42. A. Homsy, V. Linder, F. Lucklum, and N. F. de Rooij, *Sensors and Actuators B: Chemical*, **2007**. 123(1): p. 636-646 DOI: https://doi.org/10.1016/j.snb.2006.09.026.
43. M. Nakano, S. Katsura, G. G. Touchard, K. Takashima, and A. Mizuno, *IEEE Transactions on Industry Applications*, **2007**. 43(1): p. 232-237 DOI: 10.1109/TIA.2006.885899.
44. S. McNamara and Y. B. Gianchandani, *Journal of Microelectromechanical Systems*, **2005**. 14(4): p. 741-746 DOI: 10.1109/JMEMS.2005.850718.
45. N. K. Gupta and Y. B. Gianchandani, *Journal of Micromechanics and Microengineering*, **2011**. 21(9): p. 095029 DOI: 10.1088/0960-1317/21/9/095029.
46. Y.-L. Han and E. P. Muntz, *Journal of Vacuum Science & Technology B: Microelectronics and Nanometer Structures Processing, Measurement, and Phenomena*, **2007**. 25(3): p. 703-714 DOI: 10.1116/1.2723755.
47. K. Pharas and S. McNamara. *Bi-directional gas pump driven by a thermoelectric material*. in *2010 IEEE 23rd International Conference on Micro Electro Mechanical Systems (MEMS)*. 2010.
48. N. K. Gupta and Y. B. Gianchandani, *Microporous and Mesoporous Materials*, **2011**. 142(2): p. 535-541 DOI: https://doi.org/10.1016/j.micromeso.2010.12.042.
49. K. Pharas and S. McNamara, *Journal of Micromechanics and Microengineering*, **2010**. 20(12): p. 125032 DOI: 10.1088/0960-1317/20/12/125032.
50. S. Zhao, B. Jiang, T. Maeder, P. Muralt, N. Kim, S. K. Matam, E. Jeong, Y.-L. Han, and M. M. Koebel, *ACS Applied Materials & Interfaces*, **2015**. 7(33): p. 18803-18814 DOI: 10.1021/acsami.5b05462.
51. S. Zhao, G. Siqueira, S. Drdova, D. Norris, C. Ubert, A. Bonnin, S. Galmarini, M. Ganobjak, Z. Pan, S. Brunner, G. Nyström, J. Wang, M. M. Koebel, and W. J. Malfait, *Nature*, **2020**. 584(7821): p. 387-392 DOI: 10.1038/s41586-020-2594-0.
52. Y. Yang, Y. Li, M. Mao, M. Zeng, and X. Zhao, *ACS Applied Materials & Interfaces*, **2017**. 9(3): p. 2350-2357 DOI: 10.1021/acsami.6b12819.
53. J. Hou, Y. Li, M. Mao, Y. Yue, G. N. Greaves, and X. Zhao, *Nanoscale*, **2015**. 7(6): p. 2633-40 DOI: 10.1039/c4nr06410k.
54. K. A. M. Ahmed, H. Peng, K. Wu, and K. Huang, *Chemical engineering journal*, **2011**. 172(1): p. 531-539 DOI: https://doi.org/10.1016/j.cej.2011.05.070.
55. J. Zia, E. S. Aazam, and U. Riaz, *Journal of Molecular Structure*, **2020**. 1207: p. 127790 DOI: https://doi.org/10.1016/j.molstruc.2020.127790.
56. X. Xu, X. Zhou, X. Li, F. Yang, B. Jin, T. Xu, G. Li, and M. Li, *Materials Research Bulletin*, **2014**. 59: p. 32-36 DOI: https://doi.org/10.1016/j.materresbull.2014.06.025.
57. M. C. Nevarez-Martinez, M. P. Kobylanski, P. Mazierski, J. Wolkiewicz, G. Trykowski, A. Malankowska, M. Kozak, P. J. Espinoza-Montero, and A. Zaleska-Medynska, *Molecules*, **2017**. 22(4) DOI: 10.3390/molecules22040564.
58. P. Wu, S. Dai, G. Chen, S. Zhao, Z. Xu, M. Fu, P. Chen, Q. Chen, X. Jin, and Y. Qiu, *Applied Catalysis B: Environmental*, **2020**. 268: p. 118418 DOI: https://doi.org/10.1016/j.apcatb.2019.118418.
59. E. Yu, J. Chen, and H. Jia, *Journal of Hazardous Materials*, **2020**. 399: p. 122942 DOI: https://doi.org/10.1016/j.jhazmat.2020.122942.

60. J. Zhou, M. Wu, Y. Zhang, C. Zhu, Y. Fang, Y. Li, and L. Yu, *Applied Surface Science*, **2018**. 447: p. 191-199 DOI: https://doi.org/10.1016/j.apsusc.2018.03.183.
